# Comprehensive Pan-Cancer Analysis of Heat Shock Protein 110, 90, 70, and 60 Families

**DOI:** 10.3389/fmolb.2021.726244

**Published:** 2021-10-12

**Authors:** Li-rong Yan, Shi-xuan Shen, Ang Wang, Han-xi Ding, Ying-nan Liu, Yuan Yuan, Qian Xu

**Affiliations:** Tumor Etiology and Screening Department of Cancer Institute and General Surgery, The First Affiliated Hospital of China Medical University, Key Laboratory of Cancer Etiology and Prevention, China Medical University, Liaoning Provincial Education Department, Shenyang, China

**Keywords:** heat shock proteins, expression, prognosis, mutation, pan-cancer

## Abstract

**Background:** Here we carried out a panoramic analysis of the expression and prognosis of HSP110, HSP90, HSP70, and HSP60 families in 33 types of cancer, with the aim of deepening the systematic understanding of heat shock proteins (HSPs) in cancer.

**Materials and Methods:** Next-generation sequencing data of multiple tumors were downloaded from TCGA, CCLE and Oncomine databases. RStudio 3.6.1 was used to analyze HSP110, HSP90, HSP70 and HSP60 families based on their expression in 33 types of cancer. The validations *in vivo* (stomach adenocarcinoma and colon adenocarcinoma tissues) were performed by qRT-PCR.

**Results:** HSPs were differentially expressed in different cancers. The results revealed mainly positive correlations among the expressions of HSPs in different cancers. Expressions of HSP family members were generally associated with poor prognosis in respiratory, digestive, urinary and reproductive system tumors and associated with good prognosis in cholangiocarcinoma, pheochromocytoma and paraganglioma. TCGA mutation analysis showed that HSP gene mutation rate in cancers was 0–23%. CCLE mutation analysis indicated that HSP gene mutation rate in 828 cell lines from 15 tumors was 0–17%. CNV analysis revealed that HSPs have different degrees of gene amplifications and deletions in cancers. Gene mutations of 15 HSPs influenced their protein expressions in different cancers. Copy number amplifications and deletions of 22 HSPs also impacted protein expression levels in pan-cancer. HSP gene mutation was generally a poor prognosis factor in cancers, except for uterine corpus endometrial carcinoma. CNVs in 14 HSPs showed varying influences on survival status in different cancers. HSPs may be involved in the activation and inhibition of multiple cancer-related pathways. HSP expressions were closely correlated with 22 immune cell infiltrations in different cancers. The qRT-PCR validation results *in vivo* showed that HSPA2 was down-regulated in stomach adenocarcinoma and colon adenocarcinoma; HSPA7 and HSPA1A also were down-regulated in colon adenocarcinoma. HSPA2-HSPA7 (r = 0.031, *p* = 0.009) and HSPA1A-HSPA7 (r = 0.516, *p* < 0.001) were positive correlation in colon adenocarcinoma.

**Conclusion:** These analysis and validation results show that HSP families play an important role in the occurrence and development of various tumors and are potential tumor diagnostic and prognostic biomarkers as well as anti-cancer therapeutic targets.

## Introduction

The global cancer incidence and mortality rates have risen rapidly in the 21st century, and cancer has become the main cause of death in many countries around the world (Bray et al., 2018). The main causes of the high mortality rate of cancer lie in the unknown pathogenic mechanisms and the lack of effective treatment protocols. Therefore, exploring the pathogenic mechanism of cancer is critical to develop strategies for cancer treatment.

Heat shock proteins (HSPs) are a class of molecular chaperones that help protein folding and maintain the normal structures and functions of proteins (Wu et al., 2017). HSPs protect cells from physical and chemical stimuli and stress, such as ischemia, hypoxia, high temperature, metabolic factors, alcohol and drugs, thus maintaining cell homeostasis (Tsukahara et al., 2000; Macario and Conway de Macario, 2007). On the basis of their molecular weight, HSPs are divided into different families, including the HSP110(HSPH) family, HSP90 family, HSP70 (HSPA) family, HSP60 family, HSP40 (DNAJ) family and small heat shock proteins (Kampinga et al., 2009; Wu et al., 2017). Some research has suggested that different families not only independently regulate the structures and functions of proteins, but also collaborate with each other. For instance, protein folding and degradation are synergistically regulated by the HSP70 and HSP90 families (Kravats et al., 2018; Bhattacharya et al., 2020). The HSP60 precursor is transformed into mature HSP60 complexes under the cooperating of HSP70 and HSP10 (Böttinger et al., 2015). HSP family functions and jointly role was critical to sustain homeostasis.

Multiple studies have indicated that HSPs are involved in the occurrence and development of tumors. HSPs modulate cell proliferation, angiogenesis, and the migration, invasion and metastasis of tumor cells, as well as the resistance of tumors to anti-cancer drugs (Xu et al., 2006; Wong et al., 2008; Tsai et al., 2009; Lin et al., 2010; Gibert et al., 2013; Yun et al., 2019). Several studies have also suggested HSPs as potential diagnostic and prognostic biomarkers as well as therapeutic targets of tumors (Xu et al., 2006; Wong et al., 2008; Tsai et al., 2009; Lin et al., 2010; Gibert et al., 2013; Yun et al., 2019). In addition, mutations in HSPs may have an influence on cancer risk and prognosis (Medhi et al., 2013; He et al., 2014; Zou et al., 2020). In conclusion, HSPs play an important role in tumor progression when the body is in an unbalanced state of tumorigenesis.

At present, no study has performed a panoramic analysis of HSPs in 33 types of cancer. In this study, we used The Cancer Genome Atlas (TCGA), Cancer Cell Line Encyclopedia (CCLE), Oncomine and The Human Protein Atlas (THPA) databases to analyze the expressions, mutations, copy number variations (CNVs) and prognosis profiles of HSP110, HSP90, HSP70 and HSP60 families in cancer. We investigated the potential correlation of HSP expressions with cancer-related pathways, immune cell infiltration, and prognosis in pan-cancer, as well as the relationship between HSP mutations and CNVs with HSP expressions. Stomach adenocarcinoma and colon adenocarcinoma tissues were used to validate the differentially expressed of HSPs *in vivo* by quantitative real-time polymerase chain reaction (qRT-PCR). Our study aims include analyzing HSP profiles in different cancers, deepening HSP systematic recognitions, finding potential diagnostic and prognostic biomarkers as well as anti-cancer therapeutic targets and providing important clues for further mechanism research.

## Materials and Methods

### Data Collection at Tissue, Protein and Cell Levels

The HSP expressions, mutations and CNV data in 33 types of cancer were downloaded from TCGA database (http://cancergenome.nih.gov/). Clinical information, such as survival status and survival period, was downloaded from UCSC XENA (https://xenabrowser.net/). The details of 33 types of cancer collected from TCGA were as follows: ACC, Adrenocortical carcinoma; BLCA, Bladder Urothelial Carcinoma; BRCA, Breast invasive carcinoma; CESC, Cervical squamous cell carcinoma and endocervical adenocarcinoma; CHOL, Cholangiocarcinoma; COAD, Colon adenocarcinoma; DLBC, Lymphoid Neoplasm Diffuse Large B-cell Lymphoma; ESCA, Esophageal carcinoma; GBM, Glioblastoma multiforme; HNSC, Head and Neck squamous cell carcinoma; KICH, Kidney Chromophobe; KIRC, Kidney renal clear cell carcinoma; KIRP, Kidney renal papillary cell carcinoma; LAML, Acute Myeloid Leukemia; LGG, Brain Lower Grade Glioma; LIHC, Liver hepatocellular carcinoma; LUAD, Lung adenocarcinoma; LUSC, Lung squamous cell carcinoma; MESO, Mesothelioma; OV, Ovarian serous cystadenocarcinoma; PAAD, Pancreatic adenocarcinoma; PCPG, Pheochromocytoma, and Paraganglioma; PRAD, Prostate adenocarcinoma; READ, Rectum adenocarcinoma; SARC, Sarcoma; SKCM, Skin Cutaneous Melanoma; STAD, Stomach adenocarcinoma; TGCT, Testicular Germ Cell Tumors; THCA, Thyroid carcinoma; THYM, Thymoma; UCEC, Uterine Corpus Endometrial Carcinoma; UCS, Uterine Carcinosarcoma; UVM, Uveal Melanoma. In addition, expression data of HSP mRNA levels in different cancers were downloaded from the Oncomine database (Rhodes et al., 2007). Immunohistochemistry data of HSPs in 16 tumors were collected from THPA database (https://www.proteinatlas.org/). The HSP expressions and mutations data of 828 cell lines from 15 tumors were downloaded from the CCLE database (https://portals.broadinstitute.org/ccle) (Ghandi et al., 2019).

## Comprehensive Analysis of HSP Expressions in Pan-Cancer

### Analysis of HSP mRNA Levels in Pan-Cancer Tissues and Cells

Deseq2 R package was used to analyze expression data in TCGA to identify differentially expressed HSP family members in pan-cancer tissues. *p* < 0.05 was considered statistically significant. The Oncomine database was applied to verify the differentially expressed HSP mRNAs in different cancer tissues. The cut-off criteria were *p* < 0.05, |log2 (fold change) | ≥ 2 and top 10% gene rank.

We further examined the expression profiles of HSPs in different tumor cell lines using the CCLE database. Kruskal–Wallis rank test was used to compare expressions of HSPs in pan-cancer cell lines. *p* < 0.05 was considered statistically significant.

### Analysis of HSP Protein Levels in Pan-Cancer Tissues

Immunohistochemistry data of HSPs were collected from THPA database to evaluate their expression profiles at protein level in pan-cancer. The cancer types were as follows: glioma, lung cancer, colorectal cancer, testis cancer, renal cancer, head and neck cancer, stomach cancer, pancreatic cancer, lymphoma, ovarian cancer, skin cancer, breast cancer, liver cancer, endometrial cancer, melanoma, thyroid cancer.

### Correlation Analysis Between HSP Expressions and Cancer-Related Pathways

In this study, gene set variation analysis (GSVA), an algorithm of starting from gene expression and multiple pathway information, was used to estimate the changes in pathway activity of different samples in an unsupervised manner (Hänzelmann et al., 2013; Kim et al., 2018; Zhang et al., 2020a). Univariate Pearson correlation analysis was used to calculate and determine the correlation of HSPs expression with the activation or inhibition of pathways (Zhang et al., 2020a). | r | ≥ 0.2 and *p* < 0.05 were regarded as cut-off criteria and Cytoscape 3.7.1 was applied for visualization. Further, the same method was used to analyze the correlation of HSPs expression with pathway activation in single common types of cancer, including stomach adenocarcinoma, lung adenocarcinoma, lung squamous cell carcinoma, colon adenocarcinoma, liver hepatocellular carcinoma and rectum adenocarcinoma.

### Correlation Analysis Between HSP Expressions and Immune Cell Infiltration in Pan-Cancer

CIBERSORT and Spearman correlation analysis was performed to evaluate the relationships of HSP family expression with infiltration of 22 immune cell types (Diboun et al., 2006; Newman et al., 2015; Miao et al., 2020). The cut-off value was |r | ≥ 0.3 and *p* < 0.05. RStudio 3.6.1 was used for data analysis.

### Correlation and Interaction of HSP Expressions in Pan-Cancer

HSP family members often cooperate to exert their cellular functions (Böttinger et al., 2015; Genest et al., 2019). We determined Pearson’s correlation coefficient of HSP expressions in different cancers using TCGA data. Ggcorrplot and ggthemes R package were used to perform detailed analysis and visualization. Search Tool for the Retrieval of Interacting Genes (STRING, https://string-db.org/) was applied to predict the network to explore the potential interaction relationships of HSP family members. Cytoscape 3.7.2 was used to perform analysis and visualization. Degree score was determined to evaluate interaction strength among HSPs.

### Correlation Analysis Between HSP Family Expression and Prognosis in Pan-Cancer

We analyzed the correlation of HSPs expressions with prognosis using gene expression and clinical information in TCGA. Patients were divided into two groups according to the median expression of HSPs and log-rank test was performed. *p* < 0.05 was considered to be statistically significant.

## Analysis of HSP Gene Mutations and CNVs in Pan-Cancer Tissues and Cells

### HSP Gene Mutations in Pan-Cancer Tissues and Cells

Mutation data were downloaded from TCGA and CCLE databases. RStudio 3.6.1 was used to calculate the gene mutation frequency of HSP molecules in tissues and cell lines. The mutation frequency was defined as mutation proportion in each type of cancer.

### HSP Gene CNVs in Pan-Cancer Tissues

CNV data were downloaded from TCGA database. RStudio 3.6.1 was used to calculate the copy number amplification and deletion frequency in tissues. The CNV frequency was defined as CNV proportion in each type of cancer.

### Correlation Analysis Between HSP Gene Mutations and CNVs With Expressions in Pan-Cancer

Mann–Whitney *U* test was applied to statistically identify the influence of gene mutations and CNVs on HSP expressions in pan-cancer. *p* < 0.05 was considered to be statistically significant. All analyses were performed by RStudio 3.6.1.

### Correlation Analysis Between HSP Gene Mutations and CNVs With Prognosis in Pan-Cancer

Patients were divided into two groups according to the median variation frequency of HSPs to identify the influence of mutations and CNVs on overall survival in pan-cancer. Log-rank test was performed by RStudio 3.6.1. *p* < 0.05 was considered to be statistically significant.

## qRT-PCR Validations *In Vivo*

*In vivo* validation, we employed 53 pairs stomach adenocarcinoma tissues and 42 pairs colon adenocarcinoma tissues collected from the First Affiliated Hospital of China medical university. Our research was implemented according to the Declaration of Helsinki and supported by the research ethics committee of the First Affiliated Hospital of China medical university. The written informed consents of our study were signed by all patients before the samples were collected. qRT-PCR was performed by SuperReal PreMix Plus (SYBR Green, TIANGEN). The measurements were normalized using the β-actin. The primers sequences were listed in Supplementary Table S1. SPSSv25.0 (IBM, SPSS, and Chicago, IL, United States) and GraphPad Prism V8.0 (GraphPad software, United States) were utilized to perform data analysis, and 2^−ΔCt^ was used to calculate relative expression. The differential expression profiles were assessed by Student’s t-test for normally distributed data while rank sum test for skewed distribution data. Spearman correlation analysis was used to analyze the association among genes. A Chi-square test was utilized to analyze the association of HSPs expression with clinicopathological parameters. *p* < 0.05 has statistically significant.

## Results

### HSP Expression Profiles in Pan-Cancer

#### HSP mRNA Expressions in Pan-Cancer

**FIGURE 1:**
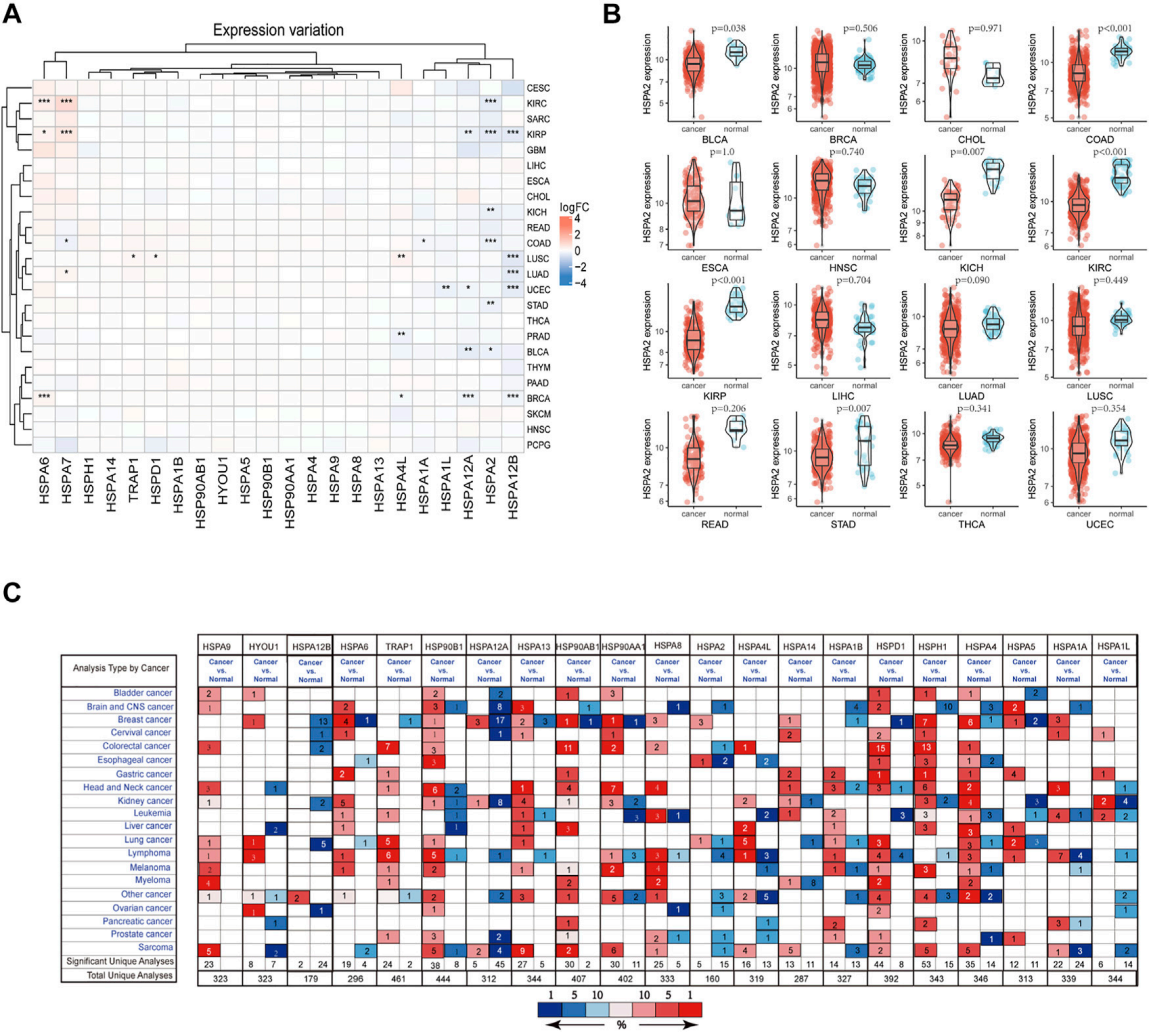
HSP expressions at mRNA level. **(A)** The differentially expressed of heat shock proteins in pan-cancer. The color in heat map represents the log2 fold change value between cancer and normal. Red color represents up-regulated and blue color represents down-regulated. *p < 0.05, **p < 0.01, ***p < 0.001. **(B)** HSPA2 expression in 16 types of cancers between cancer and normal tissues. **(C)** Expression profile of HSPs in different human cancer from Oncomine database. Red color represents up-regulated and blue color represents down-regulated. Cell color is determined by the best gene rank percentile for the analyses within the cell. HSP, heat shock protein.

On the basis of a literature review, 22 HSPs among HSP110, HSP90, HSP70 and HSP60 families were selected for analysis (Table 1) (Kampinga et al., 2009). The sample size selected per cancer type was listed in Supplementary Table S2. All results were obtained from specific TCGA platform instead of other databases in TCGA website. We examined the mRNA expression of these 22 HSPs in cancer and non-tumor tissues in TCGA and found that mRNA levels of 10 HSPs were differentially expressed in 33 types of cancer (*p* < 0.05) (Figure 1A). HSPA2 mRNA was down-regulated in stomach adenocarcinoma, colon adenocarcinoma, bladder urothelial carcinoma, kidney renal clear cell carcinoma, kidney renal papillary cell carcinoma and kidney chromophobe (Figure 1B). The mRNA expression level of HSPA7 was decreased in colon adenocarcinoma but increased in lung adenocarcinoma, kidney renal clear cell carcinoma and kidney renal papillary cell carcinoma. HSPA1A mRNA was down-regulated in colon adenocarcinoma. HSPA6 mRNA was up-regulated in breast cancer, kidney renal clear cell carcinoma and kidney renal papillary cell carcinoma. HSPA4L mRNA expression was increased in lung squamous cell carcinoma and breast cancer and decreased in prostate adenocarcinoma. HSPA1L mRNA was down-regulated in uterine corpus endometrial carcinoma. HSPA12A mRNA was down-regulated in bladder urothelial carcinoma, breast cancer, uterine corpus endometrial carcinoma and kidney renal papillary cell carcinoma. HSPA12B mRNA was down-regulated in lung squamous cell carcinoma, lung adenocarcinoma, breast cancer, uterine corpus endometrial carcinoma and kidney renal papillary cell carcinoma. The mRNA expression levels of TRAP1 and HSPD1 were increased in lung squamous cell carcinoma.

**TABLE 1:** Classifications of heat shock proteins.

Gene family	Gene name	Old names	Chrom	Chromstart	Chromend	Strand
HSP110 family	HSPH1	HSP105	chr13	31134974	31162388	−
	HSPH2	HSPA4; APG-2; HSP110	chr5	133051,962	133106449	+
	HSPH3	HSPA4L; APG-1	chr4	127781821	127840733	+
	HSPH4	HYOU1/Grp170; ORP150; HSP12A	chr11	119044189	119057202	−
HSP90 family	HSPC1	HSP90AA1; HSPN; LAP2; HSP86; HSPC1; HSPCA; HSP89; HSP90; HSP90A; HSP90N; HSPCAL1; HSPCAL4; FLJ31884	chr14	102080738	102139699	−
	HSPC3	HSP90AB1; HSPC2; HSPCB; D6S182; HSP90B; FLJ26984; HSP90-BETA	chr6	44246166	44253888	+
	HSPC4	HSP90B1; ECGP; GP96; TRA1; GRP94; endoplasmin	chr12	103930107	103953645	+
	HSPC5	TRAP1; HSP75; HSP90L	chr16	3651639	3717597	−
HSP70 family	HSPA1A	HSP70-1; HSP72; HSPA1	chr6	31815464	31817946	+
	HSPA1B	HSP70-2	chr6	31827735	31830255	+
	HSPA1L	hum70t; hum70t; Hsp-hom	chr6	31809619	31815065	−
	HSPA2	Heat-shock 70kD protein-2	chr14	64535905	64546173	+
	HSPA5	BIP; GRP78; MIF2	chr9	125234853	125241330	−
	HSPA6	Heat shock 70kD protein 6 (HSP70B′)	chr1	161524540	161526910	+
	HSPA7	Heat shock 70kD protein 7	chr1	161606291	161608217	+
	HSPA8	HSC70; HSC71; HSP71; HSP73	chr11	123057489	123063230	−
	HSPA9	GRP75; HSPA9B; MOT; MOT2; PBP74; mot-2	chr5	138554882	138575444	−
	HSPA12A	FLJ13874; KIAA0417	chr10	116671192	116742574	−
	HSPA12B	RP23-32L15.1; 2700081N06Rik	chr20	3732667	3753111	+
	HSPA13	Stch	chr21	14371115	14383484	−
	HSPA14	HSP70-4; HSP70L1; MGC131990	chr10	14838164	1,4871741	+
HSP60 family	HSPD1	HSP60; GroEL	chr2	197486581	197516737	−

We used the Oncomine database to validate the above findings. The results suggested that HSPs show varying degrees of differential expression in multiple tumors (Figure 1C).

#### HSP Protein Expressions in Pan-Cancer

**FIGURE 2:**
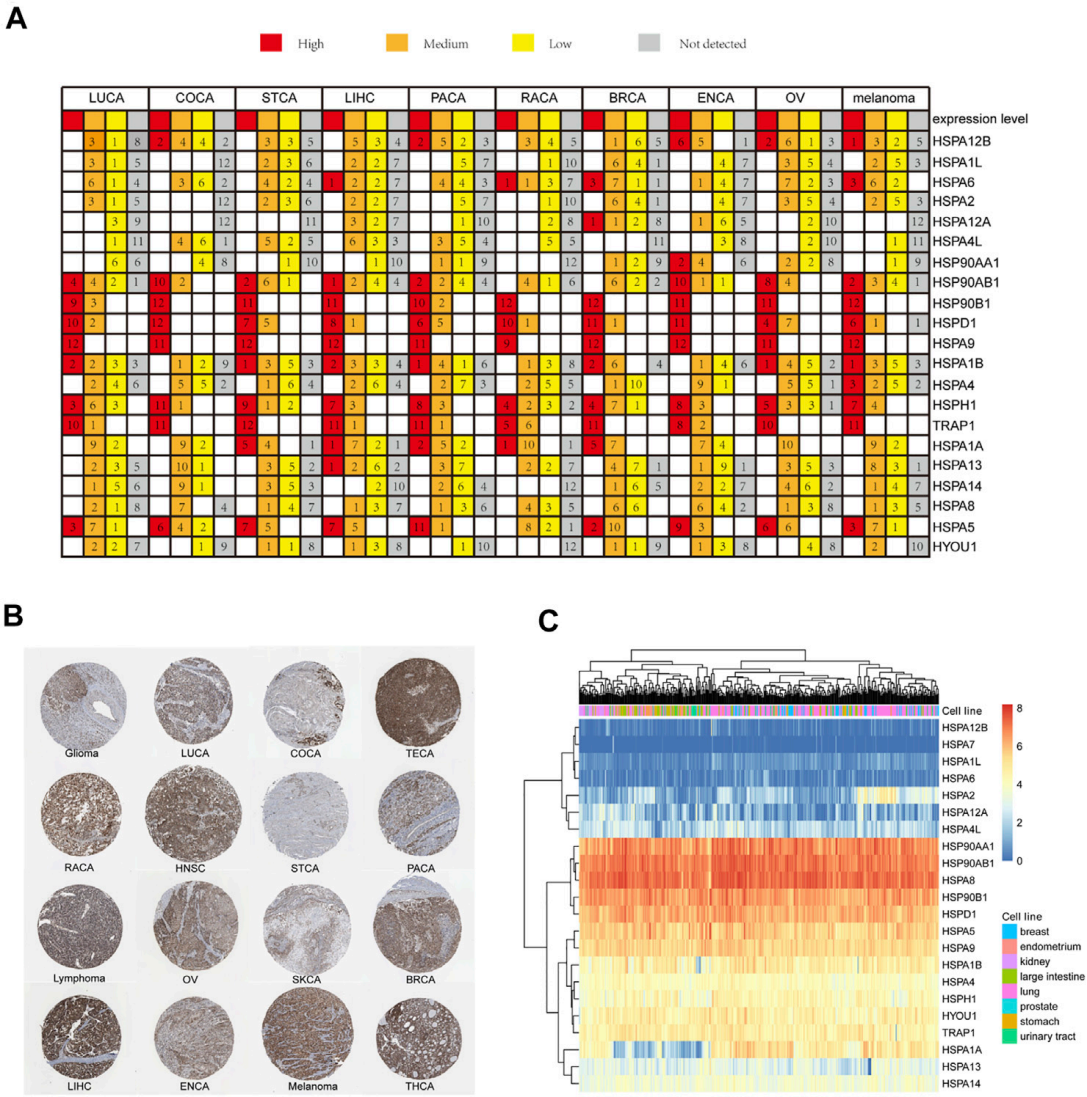
HSP expressions at protein and cell level. **(A)** HSP family members protein expression in 10 types of cancers. Each gene expression in one cancer was divided into four groups of high expression (red color), medium expression (orange color), low expression (yellow color) and not detected (grey color). **(B)** The immunohischemistry staining results of HSPA9 protein in 16 types of cancers in The Human Protein Atlas database. **(C)** Expression profile of HSPs family in cell lines in the CCLE database. Expression increased gradually from blue color to red color. CCLE, Cancer Cell Line Encyclopedia; LUCA, lung cancer; COCA, colorectal cancer; TECA, testis cancer; RACA, renal cancer; HNSC, head and neck cancer; STCA, stomach cancer; PACA; pancreatic cancer; OV, ovarian cancer; SKCA, skin cancer; BRCA, breast cancer; LIHC, liver cancer; ENCA, endometrial cancer; THCA, thyroid cancer.

We next used the THPA site to examine the expression profiles of HSP proteins in 16 tumors. Immunohistochemistry results indicated that HSP proteins showed different expression intensities in 16 tumors. The expression intensities of HSPs in 10 common tumors including lung cancer, liver cancer, colorectal cancer, pancreatic cancer, renal cancer, prostate cancer, breast cancer, endometrial cancer, ovarian cancer and melanoma were shown in Figure 2A. For example, HSP90B1, HSPA9, TRAP1, HSPH1 and HSPD1 showed high levels of immunostaining in 10 common tumors. HSPA6 showed moderate expression in lung cancer, breast cancer, endometrial cancer, ovarian cancer and melanoma and negative expression in endometrial cancer, renal cancer and liver cancer. HSPA8 showed moderate expression in other cancers. The immunostaining of HSPA9 is shown in Figure 2B.

It was found that different HSPs could be differentially expressed in 11 of 33 types of tumors at the mRNA level and 10 of 16 types of cancers at the protein level. Further, the correlation between mRNA and protein expression pattern of HSPs were concluded. The result indicated that the expression trends of partial HSPs were consistent at mRNA level and protein level in 5 types of cancer, such as TRAP1, HSPD1 and HSPA12B in lung cancer; HSPA6, HSPA12A, HSPA4L and HSPA12B in breast cancer; HSPA1L in endometrial cancer; HSPA2 and HSPA1A in colorectal cancer; HSPA12A, HSPA12B and HSPA2 in renal carcinoma (Table 2).

**TABLE 2:** The correlation of HSPs between mRNA and protein expression pattern.

Gene	Differential expression		CancerType
	mRNA level	Protein level	
HSPA6	up-regulated	up-regulated	breast cancer
TRAP1	up-regulated	up-regulated	lung cancer
HSPD1	up-regulated	up-regulated	lung cancer
HSPA2	down-regulated	down-regulated	colorectal cancer
HSPA12B	down-regulated	down-regulated	lung cancer
HSPA1L	down-regulated	down-regulated	endometrial cancer
HSPA4L	down-regulated	down-regulated	breast cancer
HSPA1A	down-regulated	down-regulated	colorectal cancer
HSPA12A	down-regulated	down-regulated	breast cancer
HSPA12A	down-regulated	down-regulated	Renal carcinoma
HSPA2	down-regulated	down-regulated	Renal carcinoma
HSPA12B	down-regulated	down-regulated	renal carcinoma
HSPA12B	down-regulated	down-regulated	breast cancer
HSPA6	up-regulated	down-regulated	Renal carcinoma
HSPA4L	up-regulated	down-regulated	lung cancer
HSPA4L	down-regulated	up-regulated	pancreatic cancer
HSPA12A	down-regulated	up-regulated	endometrial cancer
HSPA2	down-regulated	up-regulated	stomach cancer
HSPA12B	down-regulated	up-regulated	endometrial cancer

HSPs, heat shock protein.

#### HSP Expressions in Pan-Cancer Cell Lines

CCLE data revealed different expression levels of HSPs in 425 cell lines of 8 tumors, including breast cancer, endometrium cancer, kidney cancer, large intestine cancer, lung cancer, prostate cancer, stomach cancer and urinary tract cancer (Figure 2C). The following HSPs were expressed in cell lines in all eight tumors: HSP90AA1, HSP90AB1, HSPA8, HSP90B1, HSPD1, HSPA5, HSPA9, HSPA1B, HSPA4, HSPH1, HYOU1 and TRAP1. HSPA2 and HSPA1A were mainly expressed in lung cancer and breast cancer cell lines. HSPA12A was mainly expressed in lung cancer and kidney cancer cell lines. HSPA13 was mainly expressed in lung cancer, stomach cancer and endometrium cancer cell lines. HSPA14 and HSPA4L were mainly expressed in lung cancer and large intestine cancer cell lines. HSPA7, HSPA1L, HSPA12B and HSPA6 were rarely expressed in the cell lines examined.

## Relationships of HSP Expressions and Interactions in Pan-Cancer

**FIGURE 3:**
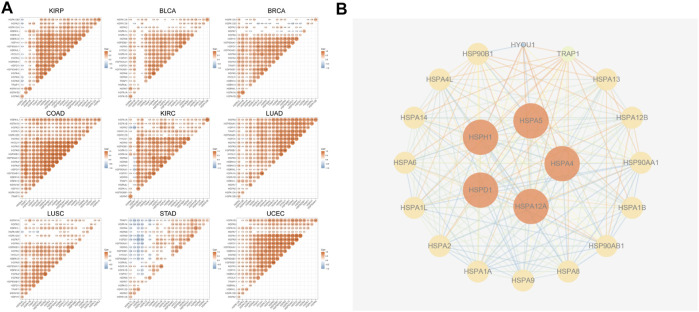
The correlation analysis and protein-protein interactions among HSPs. **(A)** The correlations among HSP expressions. Red color represents positive correlation and blue color represents negative color (*p* < 0.05). CCLE, Cancer Cell Line Encyclopedia. HSP, heat shock protein. **(B)** Protein-protein interaction network among HSP family. The higher the degree score, the larger the size.

Previous studies showed that HSPs cooperate with each other to exert critical cellular functions. Therefore, we used TCGA data to analyze the correlations of HSPs expressions in 9 tumors including stomach adenocarcinoma, breast cancer, lung adenocarcinoma, lung squamous cell carcinoma, bladder urothelial carcinoma, colon adenocarcinoma, uterine corpus endometrial carcinoma, kidney renal clear cell carcinoma and kidney renal papillary cell carcinoma. The results revealed mostly positive correlations among the expressions of HSP families in different cancers (Figure 3A), such as HSPA2-HSPA7 (r = 0.4, *p* < 0.001) and HSPA1A-HSPA7 (r = 0.397, *p* < 0.001) in colon adenocarcinoma.

We next used the STRING site to predict the potential PPI network of HSP families. The results showed that there were interactions among HSPs. HSPA4, HSPA5, HSPH1, HSPA12A and HSPD1 showed the highest degree score (Figure 3B).

## Relationships Between HSP Expressions and Cancer-Related Pathways

**FIGURE 4:**
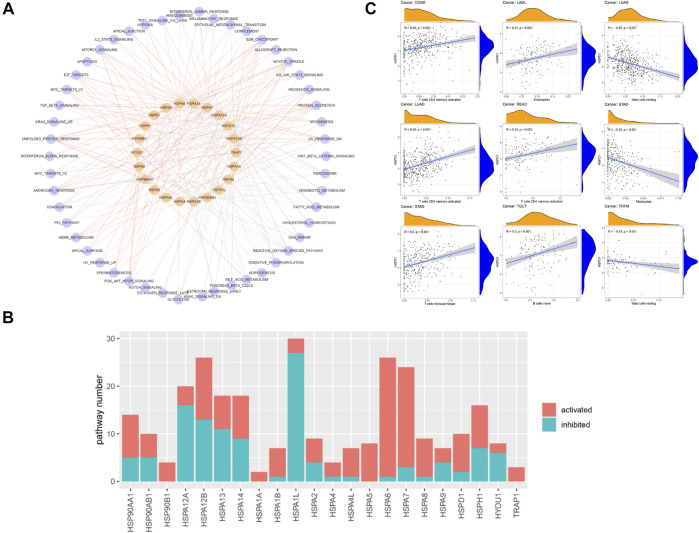
The association of HSP expressions with cancer-related pathway and immune cell infiltration. **(A)** Network displaying the correlation between HSP expressions and cancer-related pathways. Pink color represents HSPs and blue color represents pathways. The red edges represent activated pathways and grey edges represent inhibited pathways. **(B)** Number of activated and inhibited pathways of each heat shock protein. Pink color represents activated pathways activity and blue color represents inhibited pathways activity. **(C)** Association of HSPD1 with different immune cell infiltration across different cancer types. HSP, heat shock protein.

The association of HSP expressions with cancer-related pathways was analyzed. The results demonstrated that HSP family proteins mainly participate in the fatty acid metabolism pathway, oxidative phosphorylation pathway, G2M checkpoint pathway, MTORC1 signaling pathway, mitotic spindle pathway, unfolded protein response pathway, protein secretion, reactive oxygen species pathway, E2F target pathway, MYC target pathway, UV response pathway and xenobiotic metabolism pathway (Figure 4A). We counted the numbers of pathways affected by each HSP; the results suggested that HSPA1L, HSPA12B, HSPA6, HSPA7 and HSPA12A may influence the activity of most cancer-related pathways and thus potently affect tumor development (Figure 4B). In addition, the numbers of activated pathways were more than inactivated pathways affected by HSPs, indicating a cancer promotion role of these genes (Figure 4B).

Further, we analyzed the correlation of HSPs expression with pathway activation in single common types of cancer, including stomach adenocarcinoma, lung adenocarcinoma, lung squamous cell carcinoma, colon adenocarcinoma, liver hepatocellular carcinoma and rectum adenocarcinoma. The result suggested that multiple HSPs were related to the change of pathway activation in different cancers (Supplementary Table S3). In addition, we found that most HSPs have consistent conclusions in the overall and individual analyses.

## Relationships Between HSP Expressions and Immune Cell Infiltration in Pan-Cancer

Previous studies reported that HSPs participate in immune cell reactions (Todryk et al., 1950; Yamazaki et al., 2002). We next used Spearman correlation analysis to calculate the association of HSP expressions with infiltration of 22 immune cell types. HSP expressions were closely related to immune cell infiltration in the examined cancers (|r | ≥ 0.3, *p* < 0.05), except for uterine corpus endometrial carcinoma, cervical squamous cell carcinoma and endocervical adenocarcinoma, bladder urothelial carcinoma, lung squamous cell carcinoma and ovarian serous cystadenocarcinoma. The immune cell types included M1 macrophages, resting mast cells, M0 macrophages, M2 macrophages and CD4 memory activated T cells (Table 3 and Supplementary Table S4). For example, HSPD1 was positively associated with CD4 memory activated T cells and follicular helper T cells but negatively associated with monocytes in stomach adenocarcinoma, while HSPD1 was positively associated with CD4 memory activated T cells and negatively associated with resting mast cells in lung adenocarcinoma (Figure 4C).

**TABLE 3:** The relationships between heat shock protein expressions and immune cell infiltration in pan-cancer.

Gene	Cancer type	Cell type	Correlation coefficient	*p* Value	Gene	Cancer type	Cell type	Correlation coefficient	*p* Value
TRAP1	KIRP	Macrophages M0	0.35	<0.001	HSPA1A	LUAD	Macrophages M0	0.32	<0.001
TRAP1	THYM	Macrophages M1	0.34	<0.001	HSPA13	LUAD	T cells CD4 memory activated	0.36	<0.001
TRAP1	GBM	Macrophages M2	0.33	<0.001	HSPA14	LUAD	T cells CD4 memory activated	0.31	<0.001
HYOU1	LAML	Mast cells resting	0.36	<0.001	HSPA14	BRCA	T cells CD4 memory activated	0.3	<0.001
HYOU1	THYM	Macrophages M1	0.34	<0.001	HSPA14	KIRP	Macrophages M0	−0.3	<0.001
HYOU1	TGCT	Macrophages M1	0.31	<0.001	HSPA14	BRCA	Mast cells resting	−0.31	<0.001
HSPH1	STAD	Macrophages M0	0.34	<0.001	HSPA14	THYM	Macrophages M0	−0.36	<0.001
HSPH1	SARC	Mast cells resting	−0.35	<0.001	HSPA14	THYM	Macrophages M2	−0.41	<0.001
HSPH1	THYM	Mast cells resting	−0.53	<0.001	HSPA14	THYM	Mast cells resting	−0.43	<0.001
HSPH1	TGCT	Macrophages M2	−0.57	<0.001	HSPA14	ACC	Mast cells resting	−0.53	<0.001
HSPA9	KIRP	Macrophages M1	0.38	<0.001	HSPA12B	PCPG	Mast cells resting	0.42	<0.001
HSPA9	THYM	Macrophages M1	0.37	<0.001	HSPA12B	TGCT	Macrophages M2	0.39	<0.001
HSPA9	PRAD	Macrophages M1	0.31	<0.001	HSPA12B	ACC	Macrophages M2	0.35	<0.001
HSPA9	STAD	Mast cells resting	−0.35	<0.001	HSPA12B	KIRC	Mast cells resting	0.35	<0.001
HSPA8	PAAD	T cells CD4 memory activated	0.3	<0.001	HSPA12B	STAD	Mast cells resting	0.34	<0.001
HSPA8	THYM	Mast cells resting	−0.4	<0.001	HSPA12B	SARC	Macrophages M2	0.33	<0.001
HSPA7	THYM	Macrophages M1	0.41	<0.001	HSPA12B	ESCA	Mast cells resting	0.32	<0.001
HSPA7	LGG	Macrophages M1	0.32	<0.001	HSPA12B	ACC	Mast cells resting	0.31	<0.001
HSPA7	THCA	Macrophages M1	0.3	<0.001	HSPA12B	TGCT	T cells CD4 memory activated	−0.32	<0.001
HSPA6	THYM	Macrophages M2	0.45	<0.001	HSPA12B	PRAD	Macrophages M1	−0.34	<0.001
HSPA6	THYM	Macrophages M1	0.38	<0.001	HSPA12A	TGCT	Macrophages M2	0.45	<0.001
HSPA6	GBM	Macrophages M0	0.35	<0.001	HSPA12A	KIRP	Macrophages M0	−0.32	<0.001
HSPA6	SARC	T cells CD4 memory activated	0.3	<0.001	HSPA12A	KIRP	Macrophages M2	−0.32	<0.001
HSPA6	LGG	Macrophages M0	0.3	<0.001	HSPA12A	KIRP	Macrophages M0	−0.32	<0.001
HSPA6	KIRC	Mast cells resting	−0.32	<0.001	HSPA12A	KIRP	Macrophages M2	−0.32	<0.001
HSPA6	LGG	Macrophages M2	−0.33	<0.001	HSPA12A	KIRP	Macrophages M0	−0.32	<0.001
HSPA6	SARC	Mast cells resting	−0.36	<0.001	HSPA12A	KIRP	Macrophages M2	−0.32	<0.001
HSPA5	LGG	Macrophages M1	0.36	<0.001	HSPA12A	TGCT	T cells CD4 memory activated	−0.45	<0.001
HSPA5	LAML	Mast cells resting	0.36	<0.001	HSP90B1	TGCT	Macrophages M2	0.49	<0.001
HSPA5	KIRP	Macrophages M1	0.35	<0.001	HSP90B1	LGG	Macrophages M0	0.31	<0.001
HSPA5	GBM	Macrophages M0	0.31	<0.001	HSP90B1	LGG	Macrophages M1	0.31	<0.001
HSPA5	LGG	Macrophages M0	0.3	<0.001	HSP90B1	SARC	Mast cells resting	−0.3	<0.001
HSPA5	STAD	Mast cells resting	−0.3	<0.001	HSP90B1	STAD	Mast cells resting	−0.32	<0.001
HSPA5	SARC	Mast cells resting	−0.39	<0.001	HSP90AB1	KIRP	Macrophages M1	0.4	<0.001
HSPA4L	THYM	Macrophages M1	0.51	<0.001	HSP90AB1	STAD	Macrophages M0	0.35	<0.001
HSPA4L	THYM	Macrophages M2	0.4	<0.001	HSP90AB1	THYM	Macrophages M1	0.33	<0.001
HSPA4L	THYM	Macrophages M0	0.32	<0.001	HSP90AB1	TGCT	Macrophages M2	−0.33	<0.001
HSPA4L	LGG	Macrophages M1	0.3	<0.001	HSP90AB1	STAD	Mast cells resting	−0.36	<0.001
HSPA4	TGCT	T cells CD4 memory activated	0.32	<0.001	HSP90AA1	STAD	Mast cells resting	−0.3	<0.001
HSPA4	KIRP	Macrophages M1	0.3	<0.001	HSP90AA1	THYM	Mast cells resting	-0.34	<0.001
HSPA2	STAD	Mast cells resting	0.31	<0.001	HSP90AA1	TGCT	Macrophages M2	−0.37	<0.001
HSPA2	READ	T cells CD4 memory activated	−0.34	<0.001	HSPD1	THYM	Mast cells resting	−0.33	<0.001
HSPA1L	LUAD	Macrophages M0	0.32	<0.001	HSPD1	LUAD	Mast cells resting	−0.35	<0.001
HSPA1B	THYM	Macrophages M2	0.38	<0.001	HSPD1	STAD	T cells CD4 memory activated	0.32	<0.001
HSPA1B	THYM	Macrophages M0	0.36	<0.001	HSPD1	READ	T cells CD4 memory activated	0.33	<0.001
HSPA1B	THYM	Macrophages M1	0.32	<0.001	HSPD1	LUAD	T cells CD4 memory activated	0.33	<0.001
HSPA1B	LGG	Macrophages M2	−0.32	<0.001	HSPD1	COAD	T cells CD4 memory activated	0.32	<0.001

## Relationships Between HSP Expressions and Prognosis in Pan-Cancer

**FIGURE 5:**
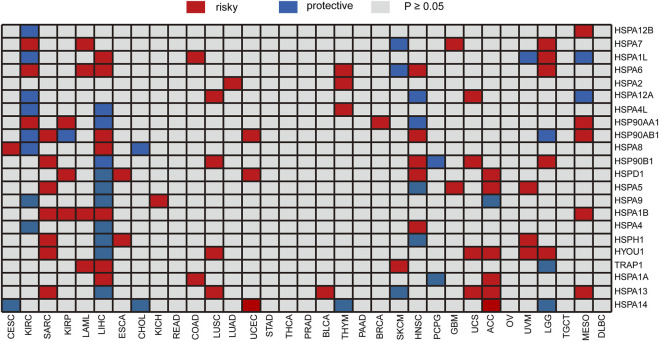
Heatmap of the relationship between individual HSP expression and overall survival. Red represents a worse prognosis, and blue represents a better prognosis. HSP, heat shock protein.

We analyzed the correlation of HSP expressions with prognosis using gene expression and clinical information in TCGA. All specimens were divided into two groups according to the median expression of HSPs, and log-rank test was performed to evaluate the correlation of expression with prognosis. The expressions of single HSP showed different effects on prognosis in 25 types of cancer, except for rectum adenocarcinoma, stomach adenocarcinoma, thymoma, prostate adenocarcinoma, pancreatic adenocarcinoma, ovarian serous cystadenocarcinoma and lymphoid neoplasm diffuse large B-cell lymphoma (Figure 5). For instance, HSPA2 was related to poor prognosis of lung adenocarcinoma and thymoma. The expression of HSPA7 hinted poor prognosis of kidney renal clear cell carcinoma, acute myeloid leukemia, brain lower grade glioma and glioblastoma multiforme, while it predicted good prognosis in skin cutaneous melanoma. HSPA1A was associated with poor prognosis of colon adenocarcinoma, liver hepatocellular carcinoma and adrenocortical carcinoma and good prognosis of pheochromocytoma and paraganglioma.

## Mutation Profiles of HSPs in Pan-Cancer Tissues and Cell Lines

**FIGURE 6:**
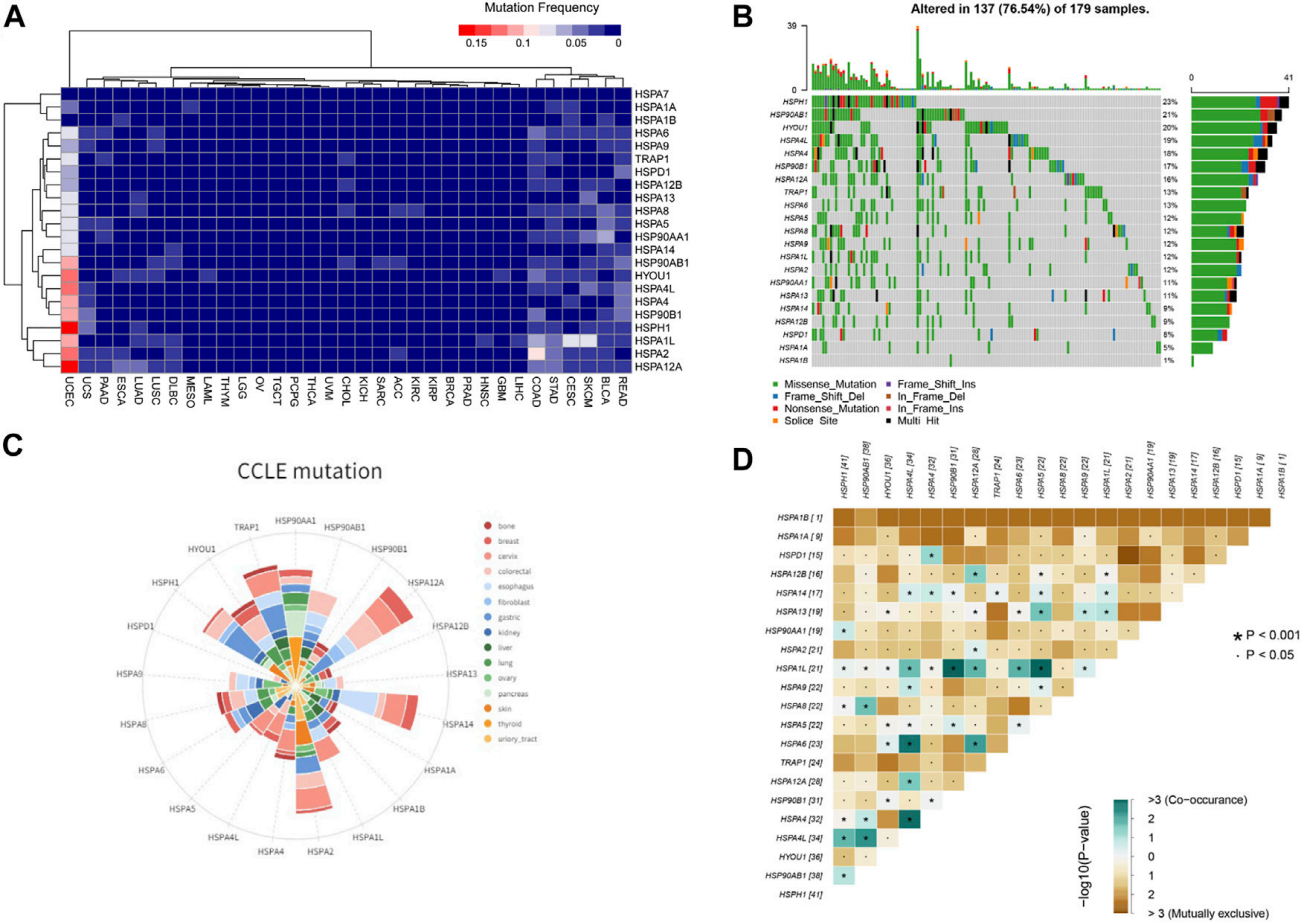
HSP mutations at tissue and cell level across different cancer types. **(A)** Mutation frequency of HSPs in tissues across different cancer types. **(B)** Oncoplot for HSPs in UCEC. HSPH1 has the most frequent mutation in UCEC. UCEC, uterine corpus endometrial carcinoma. **(C)** Mutation status of HSPs in different cancer cell lines from CCLE database. **(D)** Correlation among HSP mutations in UCEC. Blue color represents co-occurance mutation and brown color displayed mutually exclusive. The number represents sample sizes of mutation. UCEC, uterine corpus endometrial carcinoma. CCLE, Cancer Cell Line Encyclopedia. HSP, heat shock protein. UCEC, uterine corpus endometrial carcinoma.

We next examined the mutation profiles of HSPs using TCGA data. The results showed that mutations of HSPs were mainly present in uterine corpus endometrial carcinoma, colon adenocarcinoma, stomach adenocarcinoma, rectum adenocarcinoma, lung squamous cell carcinoma and lung adenocarcinoma, with a mutation frequency of 0–23% (Figure 6A). A waterfall plot for the mutation details of HSPs in uterine corpus endometrial carcinoma is shown in Figure 6B. Furthermore, CCLE analysis indicated that the mutation frequency of HSPs in 828 cell lines of 15 tumors was 0–17% (Figure 6C).

Given that the HSPs often interact synergistically in regulating cellular functions, the mutation correlation among HSP genes were analyzed in uterine corpus endometrial carcinoma of the most common mutations. The results suggested that the mutation could be co-occurred or mutually exclusive among HSPs, but the co-occurrence mutation was more obvious (Figure 6D). The co-occurrence of mutations was frequently observed in HSPA1L-HSP90B1, HSPA1L-HSPA5, HSPA4L-HSPA4 and HSPA4L-HSPA6 pairs in uterine corpus endometrial carcinoma.

**FIGURE 7:**
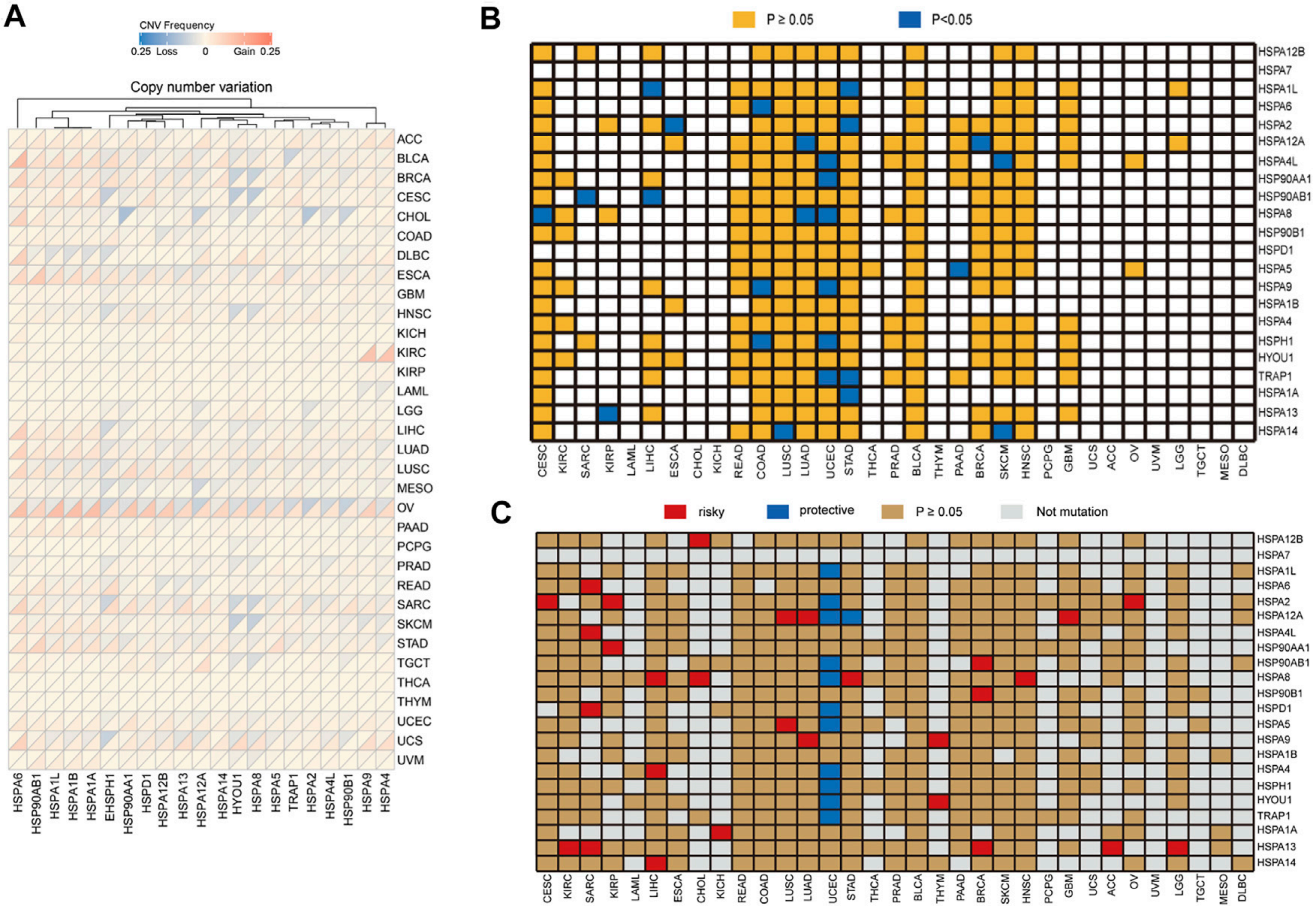
Copy number variation of HSPs and the correlation of their variation with expression and prognosis. **(A)** Copy number variation frequency of HSPs in across different cancer types. **(B)** Association of HSP mutations with expression. Orange color represents *p* ≥ 0.05, blue color represents *p* < 0.05. **(C)** Correlation between HSP mutations and prognosis in different cancer types. Red color represents a worse prognosis, blue color represents a better prognosis and brown color represents *p* ≥ 0.05. HSPs, heat shock proteins.

CNV analysis demonstrated that HSP family members showed different degrees of gene amplification and deletion in pan-cancer (Figure 7A). For instance, the gene amplification frequency of HSPA6 was 24% in bladder urothelial carcinoma and the frequency of HSP90AA1 gene deletion was 22% in cholangiocarcinoma.

## Relationships Between HSP Expressions and Gene Variations in Pan-Cancer

We then examined the correlations of HSP gene variations with expression in cancer. Our results showed that the mutation of 15 HSPs, not including HSPA7, HSPD1, HSPA4, HSPA1B, HYOU1, HSP90B1 and HSPA12B, affected their protein expression in different tumors (Table 4 and Figure 7B). Notably, the mutation of six HSPs, including HSPA4L, TRAP1, HSPH1, HSP90AA1, HSPA8 and HSPA9, was associated with their protein expression in uterine corpus endometrial carcinoma (*p* < 0.05).

**TABLE 4:** The correlation of HSPs mutations with expression and prognosis in pan-cancer.

Gene	Mutation and expression		Mutation and prognosis		CancerType
	Differential	*p* Value	HR 95% (CI)	*p* Value	
HSP90AA1	up-regulated	0.020	0.43 (0.17–1.09)	0.218	UCEC
HSPA4L	up-regulated	0.001	0.31 (0.14–0.71)	0.085	UCEC
HSPA9	up-regulated	0.043	0.30 (0.12–0.68)	0.075	UCEC
HSPA8	up-regulated	0.030	0.16 (0.07–0.37)	0.036	UCEC
HSPH1	up-regulated	0.006	0.20 (0.10–0.40)	0.013	UCEC
TRAP1	up-regulated	0.003	0	0.017	UCEC
HSPA1A	down-regulated	0.006	1.21 (0.46–3.22)	0.668	STAD
HSPA1L	down-regulated	0.008	1.48 (0.63–3.45)	0.279	STAD
HSPA2	down-regulated	0.007	1.61 (0.63–4.15)	0.210	STAD
TRAP1	up-regulated	0.031	0.84 (0.37–1.92)	0.708	STAD
HSPA12A	down-regulated	0.030	0.71 (0.14–3.76)	0.733	BRCA
HSPA8	up-regulated	0.007	0	0.386	CESC
HSPA6	up-regulated	0.022	1.02 (0.32–3.27)	0.970	COAD
HSPA9	down-regulated	0.038	0.79 (0.28–2.22)	0.685	COAD
HSPH1	down-regulated	0.047	0.84 (0.14–5.18)	0.866	COAD
HSPA2	down-regulated	0.022	0.88 (0.23–3.27)	0.851	ESCA
HSPA13	down-regulated	0.028	0	0.772	KIRP
HSP90AB1	up-regulated	0.043	0.95 (0.14–6.50)	0.961	LIHC
HSPA1L	up-regulated	0.012	2.60 (0.42–15.99)	0.090	LIHC
HSPA12A	down-regulated	<0.001	2.29 (0.68–7.66)	0.040	LUAD
HSPA8	up-regulated	0.018	1.55 (0.46–5.29)	0.380	LUAD
HSPA14	down-regulated	0.035	0	0.112	LUSC
HSPA5	up-regulated	0.019	1.98 (0.28–13.86)	0.330	PAAD
HSP90AB1	up-regulated	0.041	1.78 (0.39–8.07)	0.318	SARC
HSPA14	up-regulated	0.002	1.54 ().38–6.29)	0.453	SKCM

We further found that CNVs of HSP families impacted their expression levels in different tumors (*p* < 0.05) (Supplementary Table S5). For example, copy number amplification of HSPA2 was associated with its increased expression in breast cancer, glioblastoma multiforme, head and neck squamous carcinoma, lung squamous cell carcinoma, uterine corpus endometrial carcinoma and ovarian serous cystadenocarcinoma (Table 5). Increased copy number of HSPD1 was associated with increased expression in stomach adenocarcinoma and breast cancer, while its up-regulation was observed in pancreatic adenocarcinoma regardless of whether HSPD1 gene was amplified or deleted (Table 5).

**TABLE 5:** Relationships between heat shock protein expressions and copy number variations in pan-cancer.

Gene name	CNVCat	N	Expression median value	*p* Value	CancerType
HSPA2	DEL	62	10.303 (9.167–11.658)	0.011	BRCA
	GAIN	29	12.051 (9.649–12.666)		
	No Change	999	10.844 (9.735–12.004)		
	DEL	3	9.011 (8.298–9.706)	0.043	GBM
	GAIN	2	12.016 (11.549–12.483)		
	No Change	159	11.152 (10.204–11.958)		
	DEL	9	11.291 (9.502–12.274)	0.024	HNSC
	GAIN	25	13.069 (11.437–13.797)		
	No Change	462	12.096 (10.908–13.071)		
	DEL	43	11.47 (10.509–12.65)	0.003	LGG
	No Change	485	12.356 (11.421–13.173)		
	DEL	23	8.607 (7.803–9.212)	0.006	LUSC
	GAIN	13	10.18 (8.972–11.133)		
	No Change	464	9.377 (8.373–10.489)		
	DEL	60	9.21 (8.658–10.157)	<0.001	OV
	GAIN	23	10.852 (9.678–11.543)		
	No Change	294	10.212 (9.423–10.997)		
	DEL	7	9.264 (8.243–9.698)	0.001	UCEC
	GAIN	16	10.572 (10.139–11.177)		
	No Change	517	9.557 (8.748–10.514)		
HSPD1	DEL	19	14.242 (13.87–14.568)	<0.001	BRCA
	GAIN	60	15.065 (14.404–15.626)		
	No Change	1,011	14.287 (13.784–14.794)		
	DEL	8	13.737 (13.554–13.977)	<0.001	CESC
	GAIN	21	14.891 (14.629–15.34)		
	No Change	265	13.988 (13.442–14.525)		
	DEL	12	14.339 (13.909–14.924)	0.013	ESCA
	GAIN	10	15.273 (15.023–15.438)		
	No Change	139	14.785 (14.316–15.189)		
	DEL	27	13.661 (13.244–14.158)	<0.001	HNSC
	GAIN	26	14.896 (14.208–15.524)		
	No Change	443	14.036 (13.506–14.512)		
	DEL	11	13.422 (12.664–13.688)	0.002	KIRC
	GAIN	7	14.302 (14.146–14.692)		
	No Change	512	14.146 (13.715–14.602)		
	DEL	1	13.507 (13.507–13.507)	0.001	KIRP
	GAIN	7	15.782 (15.079–16.231)		
	No Change	279	14.106 (13.647–14.754)		
	DEL	3	14.062 (13.968–14.761)	0.029	LIHC
	GAIN	14	15.097 (14.695–15.415)		
	No Change	355	14.489 (13.979–14.991)		
	DEL	25	13.866 (13.364–14.265)	<0.001	LUSC
	GAIN	34	15.343 (15.184–15.612)		
	No Change	441	14.33 (13.791–14.855)		
	DEL	27	13.798 (13.424–14.083)	<0.001	OV
	GAIN	68	14.658 (14.153–15.103)		
	No Change	282	14.205 (13.669–14.636)		
	GAIN	6	14.156 (13.693–14.566)	0.032	PAAD
	No Change	171	13.399 (12.9–13.861)		
	DEL	4	14.354 (14.184–14.737)	0.01	PRAD
	GAIN	6	15.644 (15.17–15.92)		
	No Change	486	14.09 (13.666–14.491)		
	DEL	7	13.876 (13.827–14.053)	0.005	SKCM
	GAIN	8	15.292 (14.784–15.408)		
	No Change	455	14.426 (13.823–14.92)		
	DEL	11	14.022 (13.528–14.122)	0.007	STAD
	GAIN	14	15.382 (14.203–15.79)		
	No Change	348	14.44 (13.778–15.003)		
	DEL	6	14.111 (13.133–14.18)	0.003	UCEC
	GAIN	14	14.22 (13.973–15.407)		
	No Change	520	13.136 (12.395–14.042)		

N represented sample size.

## Relationships Between HSP Gene Variations and Prognosis in Pan-Cancer

We next examined the relationships of HSP gene variations with prognosis in pan-cancer using TCGA data. We found that mutations of HSP family genes were generally associated with good prognosis in uterine corpus endometrial carcinoma, while mutations in these genes were a poor prognostic factor in other tumors (Figure 7C). Mutations of 15 HSPs could lead to decrease or increase in expression and mutations of 20 HSPs were correlated with poor prognosis with different cancers (Table 4; Figures 7B,C). Further, we concluded and found that there were mutations of three HSPs that could cause the change of expression and were also related to prognosis (Table 4). For example, the expression of HSPA8 and HSPH1 could be increased and related to good prognosis of patients with their mutations in uterine corpus endometrial carcinoma; HSPA12A mutation in lung adenocarcinoma could down-regulate its expression and lead to poor prognosis (Table 4).

Furthermore, CNVs of HSPs, except for HSPA6, HSPA7, HSPD1, HSPA4L, HSPA14, HSPA12A, HSP90B1 and TRAP1, correlated with survival period in different cancers (Table 6). HSPA1A CNVs were correlated with shorter survival time in stomach adenocarcinoma (HR = 1.317, OR = 1.044–1.661, *p* = 0.02) and esophageal carcinoma (HR = 1.386, OR = 1.007–1.908, *p* = 0.045). HSP90AA1 CNVs was correlated with shorter survival rate in prostate adenocarcinoma (HR = 3.391, OR = 1.07–10.75, *p* = 0.038) (Table 6).

**TABLE 6:** Correlations between copy number variations and prognosis of heat shock proteins in pan-cancer.

Gene name	Variations	HR	95% CI	*p* Value	CancerType
HSP90AB1	ENSG00000096384._Del8	2.488	1.337–4.631	0.004	UCS
HSPA2	ENSG00000_Del_Gain6803.9	2.924	1.374–6.223	0.005	PAAD
HSPA8	ENSG00000_Del09971.1_Gain	29.131	2.64–321.42	0.006	KICH
HSPA12B	ENSG00000_Del3_Gain622.9	4.453	1.545–12.833	0.006	LAML
HSPA12B	ENSG00000_Del3_Gain622.9.1	4.453	1.545–12.833	0.006	LAML
HYOU1	ENSG00000_Del494_Gain8.17	29.131	2.64–321.42	0.006	KICH
HSPA5	ENSG00000044574.7	29.131	2.64–321.42	0.006	KICH
HSPA9	ENSG00000_Del13013.11	2.795	1.315–5.939	0.008	LAML
HSPA9	ENSG00000_Del13013.11	3.46	1.373–8.718	0.008	PRAD
HSPA4	ENSG00000_Del70606.1_Gain	2.795	1.315–5.939	0.008	LAML
HSPA9	ENSG00000_Del13013.11	1.927	1.154–3.217	0.012	ESCA
HSPA4	ENSG00000_Del70606.1_Gain	3.494	1.323–9.226	0.012	PRAD
HSPA1B	ENSG00000_Gain04388.6	1.317	1.044–1.661	0.02	STAD
HSPA1A	ENSG00000_Gain04389.9	1.317	1.044–1.661	0.02	STAD
HSPA1L	ENSG00000_Gain04390.9	1.317	1.044–1.661	0.02	STAD
HSPA8	ENSG00000_Del09971.1_Gain	0.587	0.371–0.927	0.022	BLCA
HSPA2	ENSG00000_Del_Gain6803.9	1.534	1.05–2.243	0.027	BRCA
HSPH1	ENSG00000_Del_Gain0694.18	0.671	0.469–0.959	0.028	LUSC
HSP90AB1	ENSG00000096384._Del8	3.645	1.097–12.108	0.035	MESO
HSP90AA1	ENSG000000808_Gain4._Del7	3.391	1.07–10.75	0.038	PRAD
HSPA4	ENSG00000_Del70606.1_Gain	1.699	1.023–2.821	0.041	CESC
HSPA5	ENSG00000044574.7	1.363	1.013–1.834	0.041	BRCA
HSPA8	ENSG00000_Del09971.1_Gain	1.944	1.019–3.709	0.044	MESO
HYOU1	ENSG00000_Del494_Gain8.17	1.986	1.02–3.867	0.044	MESO
HSPA1B	ENSG00000_Gain04388.6	1.386	1.007–1.908	0.045	ESCA
HSPA1A	ENSG00000_Gain04389.9	1.386	1.007–1.908	0.045	ESCA
HSPA1L	ENSG00000_Gain04390.9	1.386	1.007–1.908	0.045	ESCA
HSPH1	ENSG00000_Del_Gain0694.18	4.279	1.015–18.044	0.048	KIRP
HSPA13	ENSG00000_Del55304.5	1.806	1.004–3.249	0.048	CESC

HR, hazard ratio; 95%CI,95% Confidence Interval.

## qRT-PCR Validation and Clinicopathological Parameter Analysis of Differentially Expressed HSPs *In Vivo*

**FIGURE 8:**
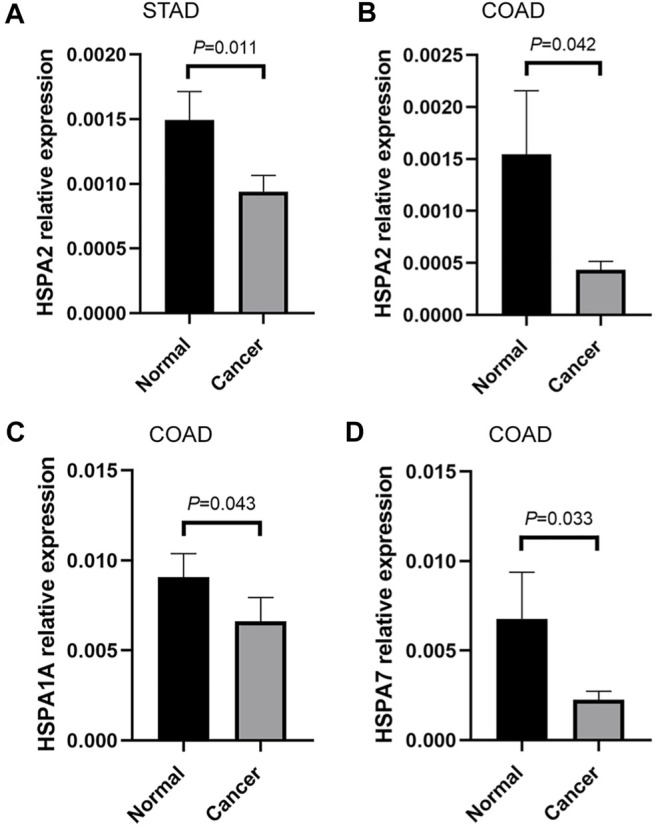
qRT-PCR validation of HSPs *in vivo*. **(A)** The mRNA relative expression of HSPA2 detected by qRT-PCR in STAD. **(B)** The mRNA relative expression of HSPA2 detected by qRT-PCR in COAD. **(C)** The mRNA relative expression of HSPA1A detected by qRT-PCR in COAD. **(D)** The mRNA relative expression of HSPA7 detected by qRT-PCR in COAD (Measurement data were expressed as mean ± SEM, *p* < 0.05). COAD, colon adenocarcinoma. HSPs, heat shock proteins. qRT-PCR, quantitative real-time polymerase chain reaction. STAD, stomach adenocarcinoma. SEM, standard error mean.

In this study, some tumors that differentially expressed HSPs, such as stomach adenocarcinoma and colon adenocarcinoma, were employed for validation *in vivo* by qRT-PCR. The result showed that HSPA2 was down-regulated in 53 pairs stomach adenocarcinoma tissues and 42 pairs colon adenocarcinoma tissues; HSPA7 and HSPA1A also were down-regulated in 42 pairs colon adenocarcinoma tissues (Figure 8). The correlation analysis also showed the significant association among genes in colon adenocarcinoma, such as HSPA2-HSPA7 (r = 0.031, *p* = 0.009), HSPA1A-HSPA7 (r = 0.516, *p* < 0.001) (Table 7). These results were consistent with above bioinformatic analysis.

**TABLE 7:** The correlation coefficient of HSPs in colon adenocarcinoma.

Gene 1	Gene 2	R	*p* Value
HSPA2	HSPA1A	0.137	0.26
HASA2	HSPA7	0.31	0.01
HSPA1A	HSPA7	0.516	<0.001

HSPs, heat shock proteins.

Further, the relationships of HSPs expression with clinicopathological parameter of gastrointestinal cancer patients were analyzed. The results showed that there is no significant association between HSPs expression and clinicopathological parameter in gastrointestinal cancer (Supplementary Tables S6, S7).

## Discussion

HSPs play important roles in various biological processes in tumor cells including cell proliferation, invasion and migration and are potential clinical biomarkers and therapeutic targets (Kumar et al., 2016; Saini and Sharma, 2018). Systematic understanding of HSP profiles in pan-cancer is helpful for us to explore its pathogenic mechanism. In this study, we downloaded the expression, survival, mutation and CNVs data in various tumors from TCGA, Oncomine, CCLE and THPA databases, which were initially used to perform a joint analysis of different levels with HSP110, HSP90, HSP70 and HSP60 families. We comprehensively combed the expression profiles of HSPs at mRNA, protein and cell levels and investigated the associations among their expressions and the correlations of expressions with cancer-related pathways, immune cell infiltration and prognosis. qRT-PCR was used to validated the differentially expressed HSPs in stomach adenocarcinoma and colon adenocarcinoma tissues.

The expression profile analysis revealed that 11 HSPs were differentially expressed in 11 tumors at the mRNA level. Furthermore, 21 HSPs showed different expression patterns at the protein level in pan-cancer. Sixteen HSPs showed high expression levels in 425 cell lines from 8 tumors. qRT-PCR validation results verified that HSPA2 was down-regulated in stomach adenocarcinoma and colon adenocarcinoma, HSPA7 and HSPA1A also were down-regulated in colon adenocarcinoma tissues. Previous studies have shown that HSPs are differentially expressed in different cancers, including HSPA2 and HSPA1A (Melendez et al., 2006; Li et al., 2014; Cao et al., 2019; Lee and Kim, 2019; Zhang et al., 2020b). Our analysis and validations indicated that HSP family members have the potential to be diagnostic biomarkers of cancer.

We further analyzed the correlation and interaction relationship among HSP family members. The results revealed a mainly positive correlation among HSP expressions in different cancers, such as HSPA2-HSPA7 and HSPA1A-HSPA7 in colon adenocarcinoma colon adenocarcinoma. Our qRT-PCR result *in vivo* also verified the significant correlation in HSPA2-HSPA7 (r = 0.031, *p* = 0.009), HSPA1A-HSPA7 (r = 0.516, *p* < 0.001) in colon adenocarcinoma tissues. This study disclosed the correlation among HSPs for the first time, which contribute to the pathogenetic mechanism research. PPI network analysis indicated that there were generally interactions among HSPs. HSPH1 interacts with HSPA5 to promote endoplasmic reticulum stress–induced caspase-3 activation and subsequent apoptosis (Meares et al., 2008). The N-terminus and C-terminus of HSPA9 interact with HSP90B1 to regulate tumor cell functions (Takano et al., 2001). Other studies showed that HSPA9 and HSPA4 influence the biological behavior of tumor cells to promote cancer progression (Gu et al., 2019; Starenki et al., 2019). This study initially found and validated the correlation and interaction among HSPs. Therefore, we speculate that these HSPs may affect tumor development by cooperating with each other. Further study is needed to explore this possibility.

Our results indicate that HSPs may be involved in the activation and inhibition of different cancer-related pathways, such as the unfolded protein response pathway, mitotic spindle pathway and reactive oxygen species pathway, demonstrating that HSPs play different functions in tumor progression. A previous study reported that HSP90 is involved in activating the IL6/JAK/STAT3 signaling pathway to affect tumor progression (Lin et al., 2013; Kolosenko et al., 2014). HSP70 inhibits oxidative phosphorylation and compensate ATP balance through enhanced glycolytic activity in HeLa cells (Wang et al., 2012). HSP90 were related to the activation of the unfolded protein response pathway in myeloma plasma cells (Davenport et al., 2007). HSPA12B secreted by tumor-associated endothelial cells induces M2 polarization of macrophages by activating the PI3K/Akt/mTOR signaling pathway, thus promoting the formation of the immunosuppressive microenvironment within tumors (Zhou et al., 2020). Our results suggested that HSPA1L, HSPA12B, HSPA12A, HSPA7, HSPH1, HYOU1, HSPA14 and HSPA13 may inhibit the oxidative phosphorylation pathway. In addition, HSPA1L, HSPA12B, HSPA12A, HSP90AB1, HSPA2, HSPA14, HSPA13 and HSPH1 may inhibit the fatty acid metabolism pathway; HSPA6, HSPH1, HSP90AA1, HSPA14, HSPA5 and HSPA8 may activate the MTORC1 signaling pathway, while HSPA1L, HSPA12B and HSPA12A may inactivate the MTORC1 signaling pathway. Previous studies have demonstrated the close correlation of tumor progression with the above pathways (Li and Zhang, 2016; Kim et al., 2017; Yu et al., 2017). Our study provides the first evidence for the association of HSPs with these cancer-related pathways. These findings may provide new clues for expanding our understanding of cancer-related pathways and their specific functions in tumor cells.

Based on previous studies demonstrating an involvement of HSPs in the immune response, we examined the correlation of HSP with immune cell infiltration. The results showed that the HSPs was related to immune cell infiltration in 28 tumors. One study reported that HSPD1 regulates the antibacterial function of neutrophils (Osterloh et al., 2009). HSP70 released by heat-stressed tumor cells induced the production of tumor cell chemokines and activation of dendritic cells via the TLR4 pathway, thus initiating anti-cancer immunity (Chen et al., 2009). M2 macrophages could elevate HSPA5 expression to trigger an inflammatory response, and thus facilitating tumor metastasis. (Zhang et al., 2017). Our results showed that HSPs were closely related to the infiltration of immune cells, such as M1 macrophages, resting mast cells, M0 macrophages, M2 macrophages and CD4 memory activated T cells. These findings may provide new directions for researching immune-targeted therapy for cancer.

Our findings showed that HSPs expressions had various effects on prognosis in 25 tumors. HSPs expressions were related to poor prognosis in several cancers, such as sarcoma, lung squamous cell carcinoma and uterine corpus endometrial carcinoma, and were associated with good prognosis in cholangiocarcinoma, pheochromocytoma and paraganglioma. HSPs expressions were correlated with poor and good prognosis in other tumors, such as liver hepatocellular carcinoma and adrenocortical carcinoma. HSPD1 promotes cell invasion and migration, which contributes to the poor prognosis in oral squamous cell carcinoma (Kang et al., 2019). The expression of HSP90AB1 also caused the worse prognosis in lung adenocarcinoma (Wang et al., 2016). HSPA9 expression was positive associated with histological grade, pathological stage and lymphatic metastasis in breast cancer, and its expression was negative associated with shorter disease-free survival and overall survival (Jin et al., 2016). Our study demonstrated that HSP expressions showed different effects on the prognosis of tumors in the digestive, respiratory, urinary and reproductive system. These results indicate that HSPs have the potential to be prognostic biomarkers of different cancers. These findings may also help establish an important foundation for further mechanism studies on how HSPs affect prognosis.

In this study, HSP expression profiles, gene mutations and CNVs as well as the correlation of gene mutations and CNVs with expression and the impact of expression on prognosis were analyzed in pan-cancer. We found that HSP mutations were frequent in uterine corpus endometrial carcinoma, colon adenocarcinoma, stomach adenocarcinoma, rectum adenocarcinoma, lung squamous cell carcinoma and lung adenocarcinoma, with a mutation frequency of 0–23%. HSP mutations were the most common in endometrial cancer, while mutations rarely occurred in testicular germ cell tumors, uveal melanoma, thymoma and thymoma. The mutation frequency of HSP genes in different cell lines of 15 tumors was 0–17%. Previous analysis illustrated that frequent mutations of HSPH1, HSPD1, HSPA4 and HSP90AA1 were detected in head and neck cancer in head and neck squamous carcinoma (Fan et al., 2020). Somatic mutations and deletions of HSPA8 were observed in sporadic breast carcinoma (Bakkenist et al., 1999). The mutation frequency of HSP90AA1 in gestational trophoblastic neoplasia was 18.2% (Luo et al., 2020). Mutations in HSP genes may play an important role in the occurrence and development of tumors and might be targets of anti-cancer treatments. The mutation correlation among HSPs was analyzed in uterine corpus endometrial carcinoma. The results showed that the co-occurrence of mutations in HSPA1L-HSP90B1, HSPA1L-HSPA5, HSPA4L-HSPA4 and HSPA4L-HSPA6 pairs were frequently observed in uterine corpus endometrial carcinoma. Therefore, we predicted that the mutations of these HSPs were closely related and might affect tumor progression.

In this study, the mutation of 15 HSPs affected protein expressions in different cancers. HSP gene mutations were generally associated with good prognosis in uterine corpus endometrial carcinoma, while mutations were poor prognostic factors in other cancers. Previous studies reported frameshift mutations of HSPA4 in gastric and colorectal cancers with microsatellite instability (Jo et al., 2016). The HSPA4 frameshift mutation seems to reduce the survival activity of tumor cells, which may partially explain why patients of gastric and colorectal cancers with microsatellite instability have better prognosis than those with microsatellite stable cancer (Jo et al., 2016). One study demonstrated that mutant HSPH1 showed disrupted cellular localization and interaction with other HSPs, thus abolishing the chaperone activity and anti-apoptotic function of HSPH1 by a dominant-negative manner (Dorard et al., 2011). In addition, HSPH1 mutation enhanced chemosensitivity to drugs, such as oxaliplatin and 5-fluorouracil, and improved prognosis in colorectal cancers with microsatellite instability (Dorard et al., 2011).

CNV analysis indicated that amplification and deletion of HSP genes in pan-cancer. Our results also showed that CNVs impacted the expression level of HSPs in different tumors. CNVs of 14 HSPs were associated with good and poor prognosis in various tumors. Somatic CNVs in general can impact protein expression level and thus influence the occurrence and development of different tumors; these CNVs have potential to be diagnostic and prognostic biomarkers as well as anti-cancer therapeutic targets (Cheau-Feng Lin et al., 2014; Hieronymus et al., 2018; Shao et al., 2019). Mutations and CNVs are crucial for tumor progression and are closely related to the pathogenic mechanisms of tumors. In our study, mutations and CNVs of HSP genes were correlated with protein expression as well as prognosis in various cancers. These variations are potential immunotherapeutic targets, and further exploration of pathogenic mechanisms is required.

## Conclusion

Our study analyzed the expression profiles of HSP110, HSP90, HSP70 and HSP60 families in pan-cancer, the relationships among their expression and the correlations of expression with cancer-related signal transduction pathways, immune cell infiltration and prognosis. We also examined the mutations and CNV profiles of HSPs as well as the association of expression with mutations and CNVs. We found that HSP family members are differently expressed in pan-cancer and are closely related to prognosis. Mutations and CNVs in HSPs exert various effects on expression and prognosis in different tumors. HSPs were closely related to immune cell infiltrations in different cancers. In this study, we comprehensively analyzed HSP110, HSP90, HSP70, and HSP60 families in cancer. These results expand our understanding of these proteins and clarified the potential roles for HSPs as diagnostic and prognostic biomarkers as well as anti-cancer therapeutic targets. Our findings provide promising clues for further research of the pathogenic mechanisms of tumors.

## Data Availability

The data that support the results of this article are available from the corresponding author upon reasonable request.

## Glossary

ACC: adrenocortical carcinoma
BLCA: bladder urothelial carcinoma
BRCA: breast invasive carcinoma
CCLE: cancer cell line encyclopedia
CNVs: copy number variations
CESC: cervical squamous cell carcinoma and endocervical adenocarcinoma
CHOL: cholangiocarcinoma
COAD: colon adenocarcinoma
DLBC: lymphoid neoplasm diffuse large B-cell lymphoma
ESCA: esophageal carcinoma
GBM: glioblastoma multiforme
HSPs: heat shock proteins
HNSC: head and neck squamous cell carcinoma
KICH: kidney Chromophobe
KIRC: kidney renal clear cell carcinoma
KIRP: kidney renal papillary cell carcinoma
LAML: acute myeloid leukemia
LGG: brain lower grade glioma
LIHC: liver hepatocellular carcinoma
LUAD: lung adenocarcinoma
LUSC: lung squamous cell carcinoma
MESO: mesothelioma
OV: ovarian serous cystadenocarcinoma
PAAD: Pancreatic adenocarcinoma
PCPG: pheochromocytoma, and paraganglioma
PPI: protein-protein interaction
PRAD: prostate adenocarcinoma
READ: rectum adenocarcinoma
SARC: sarcoma
SKCM: skin cutaneous melanoma
STAD: stomach adenocarcinoma
TGCT: testicular germ cell tumors
THCA: thyroid carcinoma
THYM: thymoma
TCGA: the cancer genome atlas
THPA: the human protein atlas
UCEC: uterine corpus endometrial carcinoma
UCS: uterine carcinosarcoma
UVM: uveal melanoma.
