# Supplementary material for: Comprehensive Pan-Cancer Analysis of Heat Shock Protein 110, 90, 70, and 60 Families
Source: Front Mol Biosci. 2021 Oct 12;8:726244. doi: 10.3389/fmolb.2021.726244 (PMC8546173; doi:10.3389/fmolb.2021.726244)
Supplement: Supplementary file 1 [file DataSheet1.docx]

**Supplementary Materials**

**Comprehensive pan-cancer analysis of heat shock protein 110, 90, 70 and 60 families**

Li-rong Yan^1^, Shi-xuan Shen^1^, Ang Wang^1^, Han-xi Ding^1^, Ying-nan Liu^1^, Yuan Yuan^1^*, Qian Xu^1^**

^1^Tumor Etiology and Screening Department of Cancer Institute and General Surgery, The First Affiliated Hospital of China Medical University, Key Laboratory of Cancer Etiology and Prevention, China Medical University, Liaoning Provincial Education Department, Shenyang 110001, China.

****Corresponding author:** Dr. Qian Xu, Tumor Etiology and Screening Department of Cancer Institute and General Surgery, North Nanjing Street 155#, Heping District, Shenyang110001, China Telephone：+86-024-83282153; fax: +86-024-83282383. Email：[qxu@cmu.edu.cn](mailto:qxu@cmu.edu.cn)

***Corresponding author:** Dr. Yuan Yuan, Tumor Etiology and Screening Department of Cancer Institute and General Surgery, North Nanjing Street 155#, Heping District, Shenyang110001, China Telephone：+86-024-83282153; fax: +86-024-83282383. Email：[yuanyuan@cmu.edu.cn](mailto:yuanyuan@cmu.edu.cn)

| **Table S1 The primer sequence used for qRT-PCR** | |
| --- | --- |
| **Primer name** | **Sequence** |
| HSPA7 |  |
| F | TGCAAAAGGATGAAAAGCCCG |
| R | TGGCTGAAGCTTCTTGTCGG |
| HSPA2 |  |
| F | TTGTTGGAAGTCTTTGGTATA |
| R | CATTTGCATTTATGCATTTGT |
| HSPA1A |  |
| F | AGCTGGAGCAGGTGTGTAAC |
| R | CAGCAATCTTGGAAAGGCCC |
| β-actin |  |
| F | ATGTGGCCGAGGACTTTGATT |
| R | AGTGGGGTGGCTTTTAGGATG |

| **Table S2 Basic information of 33 type of cancers from TCGA** | | | |
| --- | --- | --- | --- |
| **Cancer Type** | **Cancer Cases** | **Normal Cases** |  |
| Kidney renal clear cell carcinoma | 535 | 72 |  |
| Kidney renal papillary cell carcinoma | 289 | 32 |  |
| Kidney Chromophobe | 65 | 24 |  |
| Colon adenocarcinoma | 471 | 41 |  |
| Lung squamous cell carcinoma | 501 | 49 |  |
| Lung adenocarcinoma | 526 | 59 |  |
| Uterine Corpus Endometrial Carcinoma | 548 | 35 |  |
| Stomach adenocarcinoma | 375 | 32 |  |
| Prostate adenocarcinoma | 499 | 52 |  |
| Bladder Urothelial Carcinoma | 411 | 19 |  |
| Breast invasive carcinoma | 1104 | 113 |  |
| Cervical squamous cell carcinoma and endocervical adenocarcinoma | 306 | 3 |  |
| Cholangiocarcinoma | 36 | 9 |  |
| Esophageal carcinoma | 162 | 11 |  |
| Glioblastoma multiforme | 168 | 5 |  |
| Head and Neck squamous cell carcinoma | 502 | 44 |  |
| Liver hepatocellular carcinoma | 374 | 50 |  |
| Pancreatic adenocarcinoma | 178 | 4 |  |
| Pheochromocytoma, and Paraganglioma | 183 | 3 |  |
| Rectum adenocarcinoma | 167 | 10 |  |
| Sarcoma | 263 | 2 |  |
| Skin Cutaneous Melanoma | 471 | 1 |  |
| Thyroid carcinoma | 510 | 58 |  |
| Thymoma | 119 | 2 |  |
| Uterine Carcinosarcoma | 56 | 0 |  |
| Uveal Melanoma | 80 | 0 |  |
| Adrenocortical carcinoma | 79 | 0 |  |
| Lymphoid Neoplasm Diffuse Large B-cell Lymphoma | 48 | 0 |  |
| Testicular Germ Cell Tumors | 156 | 0 |  |
| Ovarian serous cystadenocarcinoma | 379 | 0 |  |
| Acute Myeloid Leukemia | 151 | 0 |  |
| Brain Lower Grade Glioma | 529 | 0 |  |
| Mesothelioma | 86 | 0 |  |
| TCGA, The Cancer Genome Atlas | | |  |

| **Table S3 The correlation of HSPs expressions with cancer-related pathway in different cancer.** | | | | |
| --- | --- | --- | --- | --- |
| **Pathway** | **HSPs** | **R value** | ***P* value** | **CancerType** |
| MYOGENESIS | HSPD1 | -0.62 | <0.001 | STAD |
| APICAL_JUNCTION | HSPD1 | -0.57 | <0.001 | STAD |
| KRAS_SIGNALING_DN | HSPD1 | -0.54 | <0.001 | STAD |
| UV_RESPONSE_DN | HSPD1 | -0.54 | <0.001 | STAD |
| MYOGENESIS | HSPA9 | -0.53 | <0.001 | STAD |
| UV_RESPONSE_DN | TRAP1 | -0.53 | <0.001 | STAD |
| APICAL_SURFACE | HSPD1 | -0.52 | <0.001 | STAD |
| E2F_TARGETS | HSPA2 | -0.52 | <0.001 | STAD |
| HEDGEHOG_SIGNALING | HSPD1 | -0.52 | <0.001 | STAD |
| TGF_BETA_SIGNALING | TRAP1 | -0.52 | <0.001 | STAD |
| MYOGENESIS | HSPA14 | -0.51 | <0.001 | STAD |
| MYC_TARGETS_V1 | HSPA2 | -0.50 | <0.001 | STAD |
| KRAS_SIGNALING_DN | HSPA9 | -0.50 | <0.001 | STAD |
| TGF_BETA_SIGNALING | HSPA12B | 0.50 | <0.001 | STAD |
| UNFOLDED_PROTEIN_RESPONSE | HYOU1 | 0.50 | <0.001 | STAD |
| IL2_STAT5_SIGNALING | HSPA12B | 0.50 | <0.001 | STAD |
| E2F_TARGETS | HSPA9 | 0.50 | <0.001 | STAD |
| KRAS_SIGNALING_DN | HSPA2 | 0.50 | <0.001 | STAD |
| MYC_TARGETS_V2 | HSP90AB1 | 0.51 | <0.001 | STAD |
| UV_RESPONSE_DN | HSPA12A | 0.51 | <0.001 | STAD |
| EPITHELIAL_MESENCHYMAL_TRANSITION | HSPA12A | 0.51 | <0.001 | STAD |
| E2F_TARGETS | TRAP1 | 0.51 | <0.001 | STAD |
| SPERMATOGENESIS | HSPA14 | 0.52 | <0.001 | STAD |
| HEDGEHOG_SIGNALING | HSPA2 | 0.52 | <0.001 | STAD |
| KRAS_SIGNALING_UP | HSPA12B | 0.52 | <0.001 | STAD |
| MTORC1_SIGNALING | HSP90AA1 | 0.52 | <0.001 | STAD |
| MYC_TARGETS_V1 | HSPA9 | 0.52 | <0.001 | STAD |
| EPITHELIAL_MESENCHYMAL_TRANSITION | HSPA2 | 0.53 | <0.001 | STAD |
| MYC_TARGETS_V1 | HSP90AA1 | 0.53 | <0.001 | STAD |
| DNA_REPAIR | HSPD1 | 0.54 | <0.001 | STAD |
| GLYCOLYSIS | HSPA5 | 0.54 | <0.001 | STAD |
| UNFOLDED_PROTEIN_RESPONSE | HSPA9 | 0.55 | <0.001 | STAD |
| MTORC1_SIGNALING | HSPA9 | 0.55 | <0.001 | STAD |
| E2F_TARGETS | HSPA14 | 0.55 | <0.001 | STAD |
| UV_RESPONSE_DN | HSPA12B | 0.55 | <0.001 | STAD |
| UV_RESPONSE_DN | HSPA2 | 0.56 | <0.001 | STAD |
| APICAL_JUNCTION | HSPA2 | 0.56 | <0.001 | STAD |
| MYC_TARGETS_V1 | TRAP1 | 0.56 | <0.001 | STAD |
| MTORC1_SIGNALING | HSPA5 | 0.57 | <0.001 | STAD |
| MTORC1_SIGNALING | HSP90B1 | 0.57 | <0.001 | STAD |
| DNA_REPAIR | TRAP1 | 0.57 | <0.001 | STAD |
| HEDGEHOG_SIGNALING | HSPA12A | 0.58 | <0.001 | STAD |
| HEDGEHOG_SIGNALING | HSPA12B | 0.58 | <0.001 | STAD |
| MYOGENESIS | HSPA12A | 0.58 | <0.001 | STAD |
| G2M_CHECKPOINT | HSPA14 | 0.59 | <0.001 | STAD |
| COAGULATION | HSPA12B | 0.60 | <0.001 | STAD |
| ANGIOGENESIS | HSPA12B | 0.61 | <0.001 | STAD |
| UNFOLDED_PROTEIN_RESPONSE | HSPD1 | 0.62 | <0.001 | STAD |
| UNFOLDED_PROTEIN_RESPONSE | HSP90B1 | 0.63 | <0.001 | STAD |
| UNFOLDED_PROTEIN_RESPONSE | HSPA5 | 0.63 | <0.001 | STAD |
| EPITHELIAL_MESENCHYMAL_TRANSITION | HSPA12B | 0.67 | <0.001 | STAD |
| MTORC1_SIGNALING | HSPD1 | 0.67 | <0.001 | STAD |
| G2M_CHECKPOINT | HSPD1 | 0.67 | <0.001 | STAD |
| MYOGENESIS | HSPA2 | 0.69 | <0.001 | STAD |
| E2F_TARGETS | HSPD1 | 0.69 | <0.001 | STAD |
| APICAL_JUNCTION | HSPA12B | 0.70 | <0.001 | STAD |
| MYC_TARGETS_V2 | TRAP1 | 0.70 | <0.001 | STAD |
| MYC_TARGETS_V2 | HSPD1 | 0.72 | <0.001 | STAD |
| MYOGENESIS | HSPA12B | 0.73 | <0.001 | STAD |
| MYC_TARGETS_V1 | HSPD1 | 0.75 | <0.001 | STAD |
| P53_PATHWAY | HSPA14 | -0.50 | <0.001 | LUAD |
| G2M_CHECKPOINT | HSP90AA1 | 0.50 | <0.001 | LUAD |
| MYC_TARGETS_V2 | TRAP1 | 0.51 | <0.001 | LUAD |
| DNA_REPAIR | HSPD1 | 0.51 | <0.001 | LUAD |
| UNFOLDED_PROTEIN_RESPONSE | HSP90AB1 | 0.52 | <0.001 | LUAD |
| MYC_TARGETS_V1 | HSPA9 | 0.52 | <0.001 | LUAD |
| UNFOLDED_PROTEIN_RESPONSE | HSPA5 | 0.52 | <0.001 | LUAD |
| MYC_TARGETS_V1 | HSP90AA1 | 0.52 | <0.001 | LUAD |
| SPERMATOGENESIS | HSPD1 | 0.53 | <0.001 | LUAD |
| APICAL_JUNCTION | HSPA12B | 0.54 | <0.001 | LUAD |
| HEDGEHOG_SIGNALING | HSPA12B | 0.54 | <0.001 | LUAD |
| MTORC1_SIGNALING | HSP90AA1 | 0.55 | <0.001 | LUAD |
| UNFOLDED_PROTEIN_RESPONSE | HYOU1 | 0.55 | <0.001 | LUAD |
| WNT_BETA_CATENIN_SIGNALING | HSPA12B | 0.56 | <0.001 | LUAD |
| COAGULATION | HSPA12B | 0.57 | <0.001 | LUAD |
| UNFOLDED_PROTEIN_RESPONSE | HSPA9 | 0.58 | <0.001 | LUAD |
| UNFOLDED_PROTEIN_RESPONSE | HSP90B1 | 0.62 | <0.001 | LUAD |
| G2M_CHECKPOINT | HSPD1 | 0.65 | <0.001 | LUAD |
| MYOGENESIS | HSPA12B | 0.66 | <0.001 | LUAD |
| E2F_TARGETS | HSPD1 | 0.67 | <0.001 | LUAD |
| MTORC1_SIGNALING | HSPD1 | 0.71 | <0.001 | LUAD |
| UNFOLDED_PROTEIN_RESPONSE | HSPD1 | 0.71 | <0.001 | LUAD |
| MYC_TARGETS_V2 | HSPD1 | 0.75 | <0.001 | LUAD |
| MYC_TARGETS_V1 | HSPD1 | 0.79 | <0.001 | LUAD |
| MTORC1_SIGNALING | HSPA8 | 0.50 | <0.001 | COAD |
| ALLOGRAFT_REJECTION | HSPA12B | 0.50 | <0.001 | COAD |
| G2M_CHECKPOINT | HSPA4 | 0.50 | <0.001 | COAD |
| UNFOLDED_PROTEIN_RESPONSE | HSP90AA1 | 0.50 | <0.001 | COAD |
| APOPTOSIS | HSPA12B | 0.50 | <0.001 | COAD |
| G2M_CHECKPOINT | HSPA9 | 0.51 | <0.001 | COAD |
| UNFOLDED_PROTEIN_RESPONSE | HSPA5 | 0.51 | <0.001 | COAD |
| GLYCOLYSIS | HSPA5 | 0.52 | <0.001 | COAD |
| G2M_CHECKPOINT | HSP90AA1 | 0.52 | <0.001 | COAD |
| SPERMATOGENESIS | HSP90AA1 | 0.52 | <0.001 | COAD |
| NOTCH_SIGNALING | HSPA12B | 0.52 | <0.001 | COAD |
| PROTEIN_SECRETION | HSP90B1 | 0.52 | <0.001 | COAD |
| MYC_TARGETS_V1 | HSP90AA1 | 0.52 | <0.001 | COAD |
| DNA_REPAIR | HSP90AB1 | 0.52 | <0.001 | COAD |
| ANDROGEN_RESPONSE | HSP90B1 | 0.53 | <0.001 | COAD |
| UNFOLDED_PROTEIN_RESPONSE | HSP90AB1 | 0.53 | <0.001 | COAD |
| MYC_TARGETS_V1 | HSP90AB1 | 0.53 | <0.001 | COAD |
| E2F_TARGETS | HSPA14 | 0.53 | <0.001 | COAD |
| HYPOXIA | HSPA12B | 0.53 | <0.001 | COAD |
| MYC_TARGETS_V1 | HSPA14 | 0.54 | <0.001 | COAD |
| MYC_TARGETS_V2 | HSPA9 | 0.54 | <0.001 | COAD |
| DNA_REPAIR | TRAP1 | 0.54 | <0.001 | COAD |
| E2F_TARGETS | HSPA9 | 0.55 | <0.001 | COAD |
| MYC_TARGETS_V1 | HSPA4 | 0.55 | <0.001 | COAD |
| HEDGEHOG_SIGNALING | HSPA12B | 0.55 | <0.001 | COAD |
| INFLAMMATORY_RESPONSE | HSPA12B | 0.55 | <0.001 | COAD |
| IL6_JAK_STAT3_SIGNALING | HSPA12B | 0.56 | <0.001 | COAD |
| KRAS_SIGNALING_DN | HSPA12B | 0.56 | <0.001 | COAD |
| DNA_REPAIR | HSPD1 | 0.56 | <0.001 | COAD |
| APICAL_SURFACE | HSPA12B | 0.57 | <0.001 | COAD |
| KRAS_SIGNALING_UP | HSPA12B | 0.58 | <0.001 | COAD |
| MTORC1_SIGNALING | HSP90AA1 | 0.58 | <0.001 | COAD |
| MTORC1_SIGNALING | HSPA4 | 0.58 | <0.001 | COAD |
| COMPLEMENT | HSPA12B | 0.59 | <0.001 | COAD |
| UNFOLDED_PROTEIN_RESPONSE | HSPA4 | 0.59 | <0.001 | COAD |
| OXIDATIVE_PHOSPHORYLATION | HSPA9 | 0.59 | <0.001 | COAD |
| ANDROGEN_RESPONSE | HSPA13 | 0.59 | <0.001 | COAD |
| MTORC1_SIGNALING | HSPA9 | 0.60 | <0.001 | COAD |
| MTORC1_SIGNALING | HSP90B1 | 0.60 | <0.001 | COAD |
| MYC_TARGETS_V2 | TRAP1 | 0.60 | <0.001 | COAD |
| IL2_STAT5_SIGNALING | HSPA12B | 0.62 | <0.001 | COAD |
| UNFOLDED_PROTEIN_RESPONSE | HSPA9 | 0.62 | <0.001 | COAD |
| MYC_TARGETS_V1 | HSPA9 | 0.63 | <0.001 | COAD |
| G2M_CHECKPOINT | HSPD1 | 0.63 | <0.001 | COAD |
| UNFOLDED_PROTEIN_RESPONSE | HSP90B1 | 0.63 | <0.001 | COAD |
| PROTEIN_SECRETION | HSPA13 | 0.65 | <0.001 | COAD |
| MTORC1_SIGNALING | HSPD1 | 0.66 | <0.001 | COAD |
| E2F_TARGETS | HSPD1 | 0.66 | <0.001 | COAD |
| MYC_TARGETS_V2 | HSPD1 | 0.67 | <0.001 | COAD |
| ANGIOGENESIS | HSPA12B | 0.67 | <0.001 | COAD |
| UNFOLDED_PROTEIN_RESPONSE | HSPD1 | 0.68 | <0.001 | COAD |
| APICAL_JUNCTION | HSPA12B | 0.69 | <0.001 | COAD |
| EPITHELIAL_MESENCHYMAL_TRANSITION | HSPA12B | 0.69 | <0.001 | COAD |
| COAGULATION | HSPA12B | 0.71 | <0.001 | COAD |
| MYOGENESIS | HSPA12B | 0.73 | <0.001 | COAD |
| MYC_TARGETS_V1 | HSPD1 | 0.78 | <0.001 | COAD |
| KRAS_SIGNALING_DN | HSPD1 | -0.56 | <0.001 | READ |
| APOPTOSIS | HSPA13 | 0.50 | <0.001 | READ |
| MTORC1_SIGNALING | HYOU1 | 0.50 | <0.001 | READ |
| G2M_CHECKPOINT | HSPA9 | 0.50 | <0.001 | READ |
| HYPOXIA | HSPA12B | 0.50 | <0.001 | READ |
| PI3K_AKT_MTOR_SIGNALING | HSP90B1 | 0.50 | <0.001 | READ |
| MITOTIC_SPINDLE | HSPH1 | 0.51 | <0.001 | READ |
| KRAS_SIGNALING_UP | HSPA12B | 0.51 | <0.001 | READ |
| MITOTIC_SPINDLE | HSPA5 | 0.51 | <0.001 | READ |
| MITOTIC_SPINDLE | HSP90AB1 | 0.51 | <0.001 | READ |
| ANDROGEN_RESPONSE | HSPA4 | 0.51 | <0.001 | READ |
| UNFOLDED_PROTEIN_RESPONSE | HSPA5 | 0.51 | <0.001 | READ |
| PI3K_AKT_MTOR_SIGNALING | HYOU1 | 0.51 | <0.001 | READ |
| MITOTIC_SPINDLE | HSP90B1 | 0.51 | <0.001 | READ |
| INFLAMMATORY_RESPONSE | HSPA12B | 0.52 | <0.001 | READ |
| MYC_TARGETS_V2 | HSPD1 | 0.52 | <0.001 | READ |
| UNFOLDED_PROTEIN_RESPONSE | HYOU1 | 0.53 | <0.001 | READ |
| G2M_CHECKPOINT | HSPA14 | 0.54 | <0.001 | READ |
| MITOTIC_SPINDLE | HSPA4 | 0.54 | <0.001 | READ |
| E2F_TARGETS | HSP90AB1 | 0.54 | <0.001 | READ |
| MTORC1_SIGNALING | HSPA8 | 0.54 | <0.001 | READ |
| IL2_STAT5_SIGNALING | HSPA12B | 0.55 | <0.001 | READ |
| NOTCH_SIGNALING | HSPA12B | 0.56 | <0.001 | READ |
| APICAL_SURFACE | HSPA12B | 0.56 | <0.001 | READ |
| G2M_CHECKPOINT | HSPA4 | 0.56 | <0.001 | READ |
| PROTEIN_SECRETION | HSPA5 | 0.56 | <0.001 | READ |
| E2F_TARGETS | HSPD1 | 0.56 | <0.001 | READ |
| UNFOLDED_PROTEIN_RESPONSE | HSP90AB1 | 0.57 | <0.001 | READ |
| MTORC1_SIGNALING | HSPA14 | 0.57 | <0.001 | READ |
| MTORC1_SIGNALING | HSP90AB1 | 0.58 | <0.001 | READ |
| HEDGEHOG_SIGNALING | HSPA12B | 0.59 | <0.001 | READ |
| MYC_TARGETS_V1 | HSPA4 | 0.59 | <0.001 | READ |
| MYC_TARGETS_V1 | HSP90AB1 | 0.59 | <0.001 | READ |
| MYC_TARGETS_V1 | HSPA9 | 0.59 | <0.001 | READ |
| G2M_CHECKPOINT | HSP90AB1 | 0.60 | <0.001 | READ |
| MTORC1_SIGNALING | HSP90B1 | 0.60 | <0.001 | READ |
| G2M_CHECKPOINT | HSPD1 | 0.60 | <0.001 | READ |
| E2F_TARGETS | HSPA14 | 0.60 | <0.001 | READ |
| UNFOLDED_PROTEIN_RESPONSE | HSP90B1 | 0.61 | <0.001 | READ |
| MYC_TARGETS_V2 | TRAP1 | 0.62 | <0.001 | READ |
| MYC_TARGETS_V1 | HSPA14 | 0.62 | <0.001 | READ |
| ANDROGEN_RESPONSE | HSP90B1 | 0.64 | <0.001 | READ |
| MTORC1_SIGNALING | HSPA9 | 0.64 | <0.001 | READ |
| ANDROGEN_RESPONSE | HSPA13 | 0.65 | <0.001 | READ |
| ANDROGEN_RESPONSE | HSPA5 | 0.65 | <0.001 | READ |
| UNFOLDED_PROTEIN_RESPONSE | HSPD1 | 0.65 | <0.001 | READ |
| MTORC1_SIGNALING | HSPA4 | 0.66 | <0.001 | READ |
| PROTEIN_SECRETION | HSP90B1 | 0.66 | <0.001 | READ |
| UNFOLDED_PROTEIN_RESPONSE | HSPA9 | 0.67 | <0.001 | READ |
| MTORC1_SIGNALING | HSPD1 | 0.68 | <0.001 | READ |
| PROTEIN_SECRETION | HSPA13 | 0.69 | <0.001 | READ |
| EPITHELIAL_MESENCHYMAL_TRANSITION | HSPA12B | 0.69 | <0.001 | READ |
| ANGIOGENESIS | HSPA12B | 0.69 | <0.001 | READ |
| UNFOLDED_PROTEIN_RESPONSE | HSPA4 | 0.69 | <0.001 | READ |
| APICAL_JUNCTION | HSPA12B | 0.69 | <0.001 | READ |
| COAGULATION | HSPA12B | 0.71 | <0.001 | READ |
| MYOGENESIS | HSPA12B | 0.71 | <0.001 | READ |
| MYC_TARGETS_V1 | HSPD1 | 0.74 | <0.001 | READ |
| MYC_TARGETS_V1 | HSPA4 | 0.50 | <0.001 | LIHC |
| APICAL_SURFACE | HSPA12B | 0.50 | <0.001 | LIHC |
| UNFOLDED_PROTEIN_RESPONSE | HSP90AB1 | 0.51 | <0.001 | LIHC |
| UNFOLDED_PROTEIN_RESPONSE | HSPD1 | 0.51 | <0.001 | LIHC |
| MYC_TARGETS_V2 | HSPA4 | 0.51 | <0.001 | LIHC |
| G2M_CHECKPOINT | HSPA13 | 0.53 | <0.001 | LIHC |
| ANGIOGENESIS | HSPA12B | 0.54 | <0.001 | LIHC |
| MTORC1_SIGNALING | HSPA4 | 0.54 | <0.001 | LIHC |
| MYC_TARGETS_V1 | HSP90AB1 | 0.55 | <0.001 | LIHC |
| PI3K_AKT_MTOR_SIGNALING | HSPA13 | 0.55 | <0.001 | LIHC |
| APICAL_JUNCTION | HSPA12B | 0.55 | <0.001 | LIHC |
| UNFOLDED_PROTEIN_RESPONSE | HYOU1 | 0.56 | <0.001 | LIHC |
| MYC_TARGETS_V2 | HSPD1 | 0.57 | <0.001 | LIHC |
| EPITHELIAL_MESENCHYMAL_TRANSITION | HSPA12B | 0.57 | <0.001 | LIHC |
| MYOGENESIS | HSPA12B | 0.57 | <0.001 | LIHC |
| PROTEIN_SECRETION | HYOU1 | 0.57 | <0.001 | LIHC |
| PROTEIN_SECRETION | HSPA13 | 0.61 | <0.001 | LIHC |
| UNFOLDED_PROTEIN_RESPONSE | HSPA5 | 0.62 | <0.001 | LIHC |
| MYC_TARGETS_V1 | HSPD1 | 0.64 | <0.001 | LIHC |
| MITOTIC_SPINDLE | HSPA13 | 0.67 | <0.001 | LIHC |

| **Table S4 The relationships between heat shock protein expressions and immune cell infiltration in pan-cancer** | | | | |
| --- | --- | --- | --- | --- |
| **Gene** | **CancerType** | **CellType** | **Correlation Coefficient** | ***P* value** |
| TRAP1 | CHOL | T cells CD4 memory resting | 0.57 | 0.001 |
| TRAP1 | ACC | T cells CD4 memory resting | 0.48 | 0.002 |
| TRAP1 | UVM | NK cells resting | 0.39 | 0.027 |
| TRAP1 | KICH | T cells CD4 memory resting | 0.38 | 0.022 |
| TRAP1 | DLBC | Dendritic cells resting | -0.3 | 0.040 |
| TRAP1 | ACC | T cells CD4 memory activated | -0.32 | 0.041 |
| TRAP1 | ACC | T cells follicular helper | -0.35 | 0.025 |
| TRAP1 | MESO | T cells CD4 memory activated | -0.37 | 0.001 |
| TRAP1 | UVM | B cells naive | -0.39 | 0.024 |
| TRAP1 | UVM | Plasma cells | -0.45 | 0.008 |
| TRAP1 | DLBC | T cells regulatory (T regs) | -0.47 | 0.001 |
| TRAP1 | KICH | Macrophages M2 | -0.52 | 0.001 |
| TRAP1 | KICH | Macrophages M0 | -0.54 | 0.001 |
| HYOU1 | KICH | Macrophages M1 | 0.42 | 0.012 |
| HYOU1 | DLBC | Dendritic cells activated | 0.39 | 0.006 |
| HYOU1 | DLBC | T cells CD4 memory activated | 0.35 | 0.016 |
| HYOU1 | KICH | T cells CD4 memory resting | 0.35 | 0.003 |
| HYOU1 | UCS | Macrophages M0 | 0.35 | 0.028 |
| HYOU1 | ESCA | T cells regulatory (T regs) | 0.34 | <0.001 |
| HYOU1 | KIRP | B cells naive | 0.33 | <0.001 |
| HYOU1 | PCPG | T cells regulatory (T regs) | 0.32 | 0.006 |
| HYOU1 | THYM | NK cells activated | 0.3 | 0.001 |
| HYOU1 | THYM | B cells naive | -0.33 | <0.001 |
| HYOU1 | ESCA | Dendritic cells resting | -0.35 | <0.001 |
| HYOU1 | KICH | Macrophages M0 | -0.47 | 0.004 |
| HYOU1 | UCS | Plasma cells | -0.47 | 0.003 |
| HYOU1 | KICH | Macrophages M2 | -0.48 | 0.004 |
| HSPH1 | TGCT | B cells naive | 0.6 | <0.001 |
| HSPH1 | UVM | T cells CD4 memory activated | 0.52 | 0.002 |
| HSPH1 | CHOL | Mast cells activated | 0.49 | 0.004 |
| HSPH1 | UVM | T cells follicular helper | 0.44 | 0.011 |
| HSPH1 | KICH | T cells follicular helper | 0.39 | 0.020 |
| HSPH1 | ACC | T cells CD4 memory resting | 0.36 | 0.023 |
| HSPH1 | DLBC | T cells CD4 memory resting | 0.33 | 0.021 |
| HSPH1 | PAAD | T cells CD4 memory resting | 0.31 | <0.001 |
| HSPH1 | UVM | Macrophages M1 | 0.3 | 0.026 |
| HSPH1 | MESO | Dendritic cells activated | -0.3 | 0.005 |
| HSPH1 | MESO | NK cells activated | -0.3 | 0.005 |
| HSPH1 | LAML | T cells CD8 | -0.32 | <0.001 |
| HSPH1 | THYM | NK cells activated | -0.33 | <0.001 |
| HSPH1 | UVM | Macrophages M2 | -0.36 | 0.420 |
| HSPH1 | UCS | T cells CD8 | -0.37 | 0.022 |
| HSPH1 | UVM | Plasma cells | -0.39 | 0.024 |
| HSPH1 | CHOL | Mast cells resting | -0.46 | 0.007 |
| HSPH1 | UVM | Monocytes | -0.46 | 0.007 |
| HSPH1 | CHOL | NK cells activated | -0.73 | <0.001 |
| HSPA9 | ACC | T cells CD4 memory resting | 0.57 | <0.001 |
| HSPA9 | KICH | Macrophages M1 | 0.48 | 0.003 |
| HSPA9 | UVM | T cells CD4 memory activated | 0.44 | 0.010 |
| HSPA9 | UCS | Mast cells activated | 0.43 | 0.007 |
| HSPA9 | UCS | T cells CD4 memory resting | 0.4 | 0.012 |
| HSPA9 | UVM | Macrophages M1 | 0.4 | 0.022 |
| HSPA9 | KICH | T cells CD4 memory resting | 0.38 | 0.021 |
| HSPA9 | PCPG | T cells CD4 memory resting | 0.38 | 0.001 |
| HSPA9 | KICH | T cells gamma delta | 0.37 | 0.028 |
| HSPA9 | UVM | T cells follicular helper | 0.37 | 0.033 |
| HSPA9 | ACC | Dendritic cells activated | 0.35 | 0.029 |
| HSPA9 | CHOL | Macrophages M1 | 0.35 | 0.044 |
| HSPA9 | MESO | Plasma cells | 0.32 | 0.003 |
| HSPA9 | DLBC | T cells regulatory (T regs) | -0.3 | 0.039 |
| HSPA9 | KIRP | Plasma cells | -0.3 | <0.001 |
| HSPA9 | TGCT | B cells memory | -0.31 | <0.001 |
| HSPA9 | THYM | Monocytes | -0.32 | <0.001 |
| HSPA9 | UCS | Plasma cells | -0.32 | 0.044 |
| HSPA9 | GBM | Plasma cells | -0.36 | <0.001 |
| HSPA9 | UCS | T cells CD8 | -0.37 | 0.021 |
| HSPA9 | KICH | Macrophages M2 | -0.4 | 0.018 |
| HSPA9 | KICH | Plasma cells | -0.42 | 0.012 |
| HSPA9 | ACC | T cells CD8 | -0.44 | 0.005 |
| HSPA9 | UCS | Mast cells resting | -0.44 | 0.005 |
| HSPA9 | UVM | Plasma cells | -0.45 | 0.009 |
| HSPA9 | ACC | T cells CD4 memory activated | -0.51 | 0.001 |
| HSPA9 | ACC | T cells follicular helper | -0.52 | 0.001 |
| HSPA9 | UVM | Monocytes | -0.57 | 0.001 |
| HSPA8 | ACC | Macrophages M0 | 0.44 | 0.004 |
| HSPA8 | UCS | NK cells resting | 0.4 | 0.013 |
| HSPA8 | CHOL | T cells regulatory (T regs) | 0.36 | 0.037 |
| HSPA8 | UCS | Dendritic cells resting | 0.36 | 0.023 |
| HSPA8 | KICH | T cells CD4 memory resting | 0.35 | 0.035 |
| HSPA8 | UVM | T cells CD4 memory activated | 0.35 | 0.047 |
| HSPA8 | KIRC | Neutrophils | 0.33 | <0.001 |
| HSPA8 | UCS | Mast cells activated | 0.32 | 0.047 |
| HSPA8 | READ | Neutrophils | 0.31 | <0.001 |
| HSPA8 | LUAD | Neutrophils | 0.3 | <0.001 |
| HSPA8 | PAAD | NK cells activated | -0.32 | <0.001 |
| HSPA8 | UVM | B cells memory | -0.35 | 0.043 |
| HSPA8 | ACC | Mast cells resting | -0.4 | 0.010 |
| HSPA8 | UVM | Monocytes | -0.4 | 0.021 |
| HSPA8 | ACC | B cells memory | -0.45 | 0.004 |
| HSPA7 | LAML | Monocytes | 0.56 | <0.001 |
| HSPA7 | LGG | T cells CD8 | 0.41 | <0.001 |
| HSPA7 | KICH | T cells CD8 | 0.4 | 0.015 |
| HSPA7 | UCS | Dendritic cells resting | 0.38 | 0.016 |
| HSPA7 | UCS | B cells memory | 0.34 | 0.035 |
| HSPA7 | ACC | T cells CD8 | 0.33 | 0.039 |
| HSPA7 | THYM | NK cells activated | 0.32 | <0.001 |
| HSPA7 | DLBC | T cells regulatory (Tregs) | -0.3 | 0.036 |
| HSPA7 | MESO | Dendritic cells activated | -0.3 | 0.005 |
| HSPA7 | LGG | Monocytes | -0.31 | <0.001 |
| HSPA7 | COAD | T cells CD4 memory resting | -0.32 | <0.001 |
| HSPA7 | ACC | Dendritic cells activated | -0.32 | 0.044 |
| HSPA7 | LAML | B cells naive | -0.34 | <0.001 |
| HSPA7 | UVM | Monocytes | -0.4 | 0.021 |
| HSPA7 | CHOL | Macrophages M0 | -0.46 | 0.008 |
| HSPA7 | KICH | NK cells activated | -0.47 | 0.004 |
| HSPA6 | LAML | Neutrophils | 0.57 | <0.001 |
| HSPA6 | LAML | Monocytes | 0.53 | <0.001 |
| HSPA6 | DLBC | Macrophages M1 | 0.48 | 0.001 |
| HSPA6 | ACC | T cells follicular helper | 0.45 | 0.004 |
| HSPA6 | UVM | T cells follicular helper | 0.43 | 0.012 |
| HSPA6 | UCS | Neutrophils | 0.43 | 0.007 |
| HSPA6 | DLBC | T cells CD4 memory activated | 0.41 | 0.004 |
| HSPA6 | UCS | NK cells resting | 0.41 | 0.010 |
| HSPA6 | MESO | T cells CD4 memory activated | 0.37 | 0.001 |
| HSPA6 | ACC | T cells CD4 memory activated | 0.36 | 0.023 |
| HSPA6 | UCS | Macrophages M0 | 0.36 | 0.025 |
| HSPA6 | ACC | Macrophages M1 | 0.35 | 0.026 |
| HSPA6 | TGCT | Monocytes | 0.34 | <0.001 |
| HSPA6 | CHOL | Neutrophils | 0.34 | 0.050 |
| HSPA6 | STAD | Neutrophils | 0.34 | <0.001 |
| HSPA6 | TGCT | NK cells activated | 0.31 | <0.001 |
| HSPA6 | LGG | T cells CD8 | 0.3 | <0.001 |
| HSPA6 | SARC | Monocytes | 0.3 | <0.001 |
| HSPA6 | DLBC | B cells naive | -0.31 | 0.032 |
| HSPA6 | ACC | Macrophages M2 | -0.32 | 0.044 |
| HSPA6 | LAML | T cells CD4 memory resting | -0.34 | <0.001 |
| HSPA6 | ACC | NK cells activated | -0.34 | 0.031 |
| HSPA6 | LAML | Eosinophils | -0.35 | <0.001 |
| HSPA6 | ACC | Mast cells resting | -0.37 | 0.018 |
| HSPA6 | LAML | B cells naive | -0.39 | <0.001 |
| HSPA6 | TGCT | B cells naive | -0.41 | <0.001 |
| HSPA6 | UVM | Macrophages M2 | -0.54 | 0.002 |
| HSPA5 | CHOL | T cells regulatory (Tregs) | 0.58 | <0.001 |
| HSPA5 | ACC | Macrophages M0 | 0.51 | 0.001 |
| HSPA5 | CHOL | Monocytes | 0.44 | 0.009 |
| HSPA5 | UVM | T cells CD4 memory activated | 0.42 | 0.014 |
| HSPA5 | UVM | T cells follicular helper | 0.41 | 0.018 |
| HSPA5 | ACC | Macrophages M1 | 0.39 | 0.012 |
| HSPA5 | UVM | Macrophages M1 | 0.38 | 0.028 |
| HSPA5 | UCS | Neutrophils | 0.36 | 0.024 |
| HSPA5 | KICH | Macrophages M1 | 0.35 | 0.039 |
| HSPA5 | UCS | T cells CD4 memory resting | 0.34 | 0.048 |
| HSPA5 | ACC | T cells follicular helper | 0.34 | 0.031 |
| HSPA5 | KICH | T cells CD4 memory resting | 0.33 | 0.037 |
| HSPA5 | THYM | Macrophages M1 | 0.31 | 0.001 |
| HSPA5 | THYM | Mast cells resting | 0.31 | 0.001 |
| HSPA5 | LIHC | T cells follicular helper | 0.3 | <0.001 |
| HSPA5 | PCPG | T cells regulatory (Tregs) | 0.3 | 0.009 |
| HSPA5 | THCA | Dendritic cells resting | -0.31 | <0.001 |
| HSPA5 | THCA | Dendritic cells activated | -0.34 | <0.001 |
| HSPA5 | CHOL | T cells follicular helper | -0.35 | 0.048 |
| HSPA5 | KICH | Macrophages M2 | -0.35 | 0.034 |
| HSPA5 | CHOL | T cells CD8 | -0.37 | 0.037 |
| HSPA5 | UVM | Monocytes | -0.46 | 0.007 |
| HSPA5 | UCS | Plasma cells | -0.49 | 0.001 |
| HSPA5 | UVM | Plasma cells | -0.54 | 0.001 |
| HSPA4L | KICH | T cells CD4 memory resting | 0.54 | 0.001 |
| HSPA4L | CHOL | Macrophages M1 | 0.52 | 0.002 |
| HSPA4L | CHOL | T cells CD4 memory resting | 0.51 | 0.003 |
| HSPA4L | KICH | B cells naive | 0.49 | 0.002 |
| HSPA4L | UVM | Macrophages M1 | 0.48 | 0.005 |
| HSPA4L | DLBC | Dendritic cells activated | 0.44 | 0.002 |
| HSPA4L | UVM | T cells follicular helper | 0.43 | 0.012 |
| HSPA4L | UCS | Dendritic cells resting | 0.41 | 0.010 |
| HSPA4L | UVM | T cells CD4 memory activated | 0.4 | 0.020 |
| HSPA4L | ACC | Monocytes | 0.39 | 0.013 |
| HSPA4L | THYM | NK cells activated | 0.38 | <0.001 |
| HSPA4L | UVM | T cells CD8 | 0.37 | 0.034 |
| HSPA4L | DLBC | T cells CD4 memory activated | 0.37 | 0.010 |
| HSPA4L | PCPG | Dendritic cells resting | 0.37 | 0.002 |
| HSPA4L | SARC | Dendritic cells resting | 0.36 | 0.079 |
| HSPA4L | THYM | Dendritic cells activated | 0.36 | <0.001 |
| HSPA4L | PRAD | Dendritic cells resting | 0.35 | <0.001 |
| HSPA4L | ACC | T cells CD4 memory resting | 0.34 | 0.033 |
| HSPA4L | UCS | Neutrophils | 0.33 | 0.043 |
| HSPA4L | THYM | T cells CD4 naive | 0.31 | 0.015 |
| HSPA4L | LIHC | Monocytes | -0.3 | 0.018 |
| HSPA4L | THYM | Monocytes | -0.34 | <0.001 |
| HSPA4L | ACC | Macrophages M1 | -0.34 | 0.034 |
| HSPA4L | UVM | Plasma cells | -0.35 | 0.047 |
| HSPA4L | ACC | T cells CD8 | -0.35 | 0.026 |
| HSPA4L | KIRC | T cells regulatory (Tregs) | -0.35 | <0.001 |
| HSPA4L | ACC | T cells follicular helper | -0.36 | 0.023 |
| HSPA4L | ESCA | T cells regulatory (Tregs) | -0.36 | <0.001 |
| HSPA4L | LAML | Monocytes | -0.36 | <0.001 |
| HSPA4L | UVM | Macrophages M2 | -0.36 | 0.039 |
| HSPA4L | THYM | NK cells resting | -0.38 | <0.001 |
| HSPA4L | UCS | Mast cells resting | -0.39 | 0.013 |
| HSPA4L | THYM | Dendritic cells resting | -0.43 | <0.001 |
| HSPA4L | UVM | Monocytes | -0.46 | 0.007 |
| HSPA4 | UCS | T cells CD4 memory resting | 0.5 | 0.001 |
| HSPA4 | CHOL | T cells regulatory (Tregs) | 0.47 | 0.006 |
| HSPA4 | UCS | Mast cells activated | 0.44 | 0.005 |
| HSPA4 | UVM | T cells CD4 memory activated | 0.39 | 0.026 |
| HSPA4 | KICH | Macrophages M1 | 0.35 | 0.039 |
| HSPA4 | TGCT | B cells naive | 0.31 | <0.001 |
| HSPA4 | GBM | Plasma cells | -0.3 | <0.001 |
| HSPA4 | DLBC | T cells follicular helper | -0.31 | 0.030 |
| HSPA4 | UCS | T cells CD8 | -0.33 | 0.039 |
| HSPA4 | UCS | Mast cells resting | -0.33 | 0.042 |
| HSPA4 | ACC | T cells CD8 | -0.38 | 0.015 |
| HSPA4 | ACC | Dendritic cells resting | -0.38 | 0.015 |
| HSPA4 | LAML | T cells CD8 | -0.4 | <0.001 |
| HSPA4 | KICH | Plasma cells | -0.45 | 0.006 |
| HSPA4 | UVM | Plasma cells | -0.45 | 0.009 |
| HSPA4 | ACC | T cells gamma delta | -0.46 | 0.003 |
| HSPA2 | CHOL | B cells naive | 0.52 | 0.002 |
| HSPA2 | ESCA | Dendritic cells resting | 0.45 | <0.001 |
| HSPA2 | ACC | NK cells activated | 0.42 | 0.007 |
| HSPA2 | CHOL | Plasma cells | 0.42 | 0.015 |
| HSPA2 | TGCT | Dendritic cells activated | 0.42 | <0.001 |
| HSPA2 | KICH | T cells CD8 | 0.41 | 0.013 |
| HSPA2 | CHOL | Monocytes | 0.36 | 0.037 |
| HSPA2 | THYM | B cells naive | 0.35 | <0.001 |
| HSPA2 | ESCA | T cells regulatory (Tregs) | -0.34 | <0.001 |
| HSPA2 | PCPG | T cells follicular helper | -0.37 | 0.004 |
| HSPA2 | CHOL | NK cells activated | -0.4 | 0.020 |
| HSPA2 | CHOL | B cells memory | -0.45 | 0.008 |
| HSPA2 | CHOL | Macrophages M2 | -0.53 | 0.002 |
| HSPA1L | UCS | Macrophages M0 | 0.52 | 0.001 |
| HSPA1L | KICH | Macrophages M2 | 0.51 | 0.002 |
| HSPA1L | DLBC | Macrophages M1 | 0.47 | 0.001 |
| HSPA1L | CHOL | Mast cells activated | 0.46 | 0.007 |
| HSPA1L | UCS | NK cells resting | 0.45 | 0.004 |
| HSPA1L | UVM | NK cells resting | 0.41 | 0.017 |
| HSPA1L | LAML | Monocytes | 0.37 | <0.001 |
| HSPA1L | ACC | Macrophages M1 | 0.35 | 0.026 |
| HSPA1L | LAML | Neutrophils | 0.32 | <0.001 |
| HSPA1L | THYM | Mast cells resting | 0.31 | 0.001 |
| HSPA1L | LAML | B cells naive | -0.3 | <0.001 |
| HSPA1L | UCS | Macrophages M1 | -0.33 | 0.043 |
| HSPA1L | ESCA | T cells regulatory (Tregs) | -0.34 | <0.001 |
| HSPA1L | ACC | Mast cells resting | -0.34 | 0.032 |
| HSPA1L | KICH | T cells CD4 memory resting | -0.35 | 0.036 |
| HSPA1L | UVM | T cells CD8 | -0.37 | 0.033 |
| HSPA1L | KICH | Macrophages M1 | -0.37 | 0.028 |
| HSPA1L | CHOL | B cells memory | -0.39 | 0.023 |
| HSPA1B | CHOL | Mast cells activated | 0.45 | 0.009 |
| HSPA1B | ACC | NK cells resting | 0.38 | 0.016 |
| HSPA1B | KICH | T cells regulatory (Tregs) | 0.34 | 0.043 |
| HSPA1B | UCS | T cells regulatory (Tregs) | 0.34 | 0.033 |
| HSPA1B | THYM | NK cells activated | 0.33 | <0.001 |
| HSPA1B | UCS | NK cells resting | 0.32 | 0.044 |
| HSPA1B | DLBC | Macrophages M1 | 0.3 | 0.039 |
| HSPA1B | THYM | Mast cells resting | 0.3 | 0.001 |
| HSPA1B | ESCA | T cells regulatory (Tregs) | -0.31 | <0.001 |
| HSPA1B | KICH | Eosinophils | -0.35 | 0.036 |
| HSPA1B | THYM | Dendritic cells resting | -0.38 | <0.001 |
| HSPA1B | CHOL | B cells memory | -0.42 | 0.014 |
| HSPA1A | UCS | Macrophages M0 | 0.52 | 0.001 |
| HSPA1A | KICH | Macrophages M2 | 0.51 | 0.002 |
| HSPA1A | DLBC | Macrophages M1 | 0.47 | 0.001 |
| HSPA1A | CHOL | Mast cells activated | 0.46 | 0.007 |
| HSPA1A | UCS | NK cells resting | 0.45 | 0.004 |
| HSPA1A | UVM | NK cells resting | 0.41 | 0.017 |
| HSPA1A | LAML | Monocytes | 0.37 | <0.001 |
| HSPA1A | ACC | Macrophages M1 | 0.35 | 0.026 |
| HSPA1A | LAML | Neutrophils | 0.32 | <0.001 |
| HSPA1A | THYM | Mast cells resting | 0.31 | 0.001 |
| HSPA1A | LAML | B cells naive | -0.3 | <0.001 |
| HSPA1A | UCS | Macrophages M1 | -0.33 | 0.043 |
| HSPA1A | ESCA | T cells regulatory (Tregs) | -0.34 | <0.001 |
| HSPA1A | ACC | Mast cells resting | -0.34 | 0.032 |
| HSPA1A | KICH | T cells CD4 memory resting | -0.35 | 0.036 |
| HSPA1A | DLBC | B cells naive | -0.36 | 0.013 |
| HSPA1A | UVM | T cells CD8 | -0.37 | 0.033 |
| HSPA1A | KICH | Macrophages M1 | -0.37 | 0.028 |
| HSPA1A | CHOL | B cells memory | -0.39 | 0.023 |
| HSPA14 | UVM | T cells CD4 memory activated | 0.53 | 0.002 |
| HSPA14 | UVM | T cells follicular helper | 0.49 | 0.004 |
| HSPA14 | UVM | Macrophages M1 | 0.46 | 0.007 |
| HSPA14 | ACC | Macrophages M0 | 0.45 | 0.003 |
| HSPA14 | THYM | Dendritic cells resting | 0.42 | <0.001 |
| HSPA14 | LAML | Eosinophils | 0.34 | <0.001 |
| HSPA14 | ACC | T cells follicular helper | 0.32 | 0.042 |
| HSPA14 | LIHC | T cells follicular helper | 0.31 | <0.001 |
| HSPA14 | TGCT | B cells naive | 0.3 | <0.001 |
| HSPA14 | KICH | Macrophages M2 | -0.38 | 0.024 |
| HSPA14 | UVM | Plasma cells | -0.38 | 0.028 |
| HSPA14 | ACC | T cells gamma delta | -0.4 | 0.010 |
| HSPA14 | KICH | NK cells activated | -0.41 | 0.014 |
| HSPA14 | DLBC | T cells regulatory (T regs) | -0.42 | 0.003 |
| HSPA13 | UVM | T cells CD4 memory activated | 0.48 | 0.005 |
| HSPA13 | PCPG | T cells CD4 memory resting | 0.44 | <0.001 |
| HSPA13 | UVM | T cells follicular helper | 0.42 | 0.016 |
| HSPA13 | ACC | Macrophages M0 | 0.4 | 0.012 |
| HSPA13 | DLBC | T cells CD4 memory activated | 0.35 | 0.016 |
| HSPA13 | SKCM | T cells CD4 memory resting | 0.32 | <0.001 |
| HSPA13 | THYM | T cells CD4 memory resting | 0.31 | 0.001 |
| HSPA13 | COAD | Eosinophils | 0.3 | <0.001 |
| HSPA13 | COAD | Neutrophils | 0.3 | <0.001 |
| HSPA13 | THYM | T cells CD4 memory activated | -0.3 | 0.001 |
| HSPA13 | PAAD | B cells memory | -0.31 | <0.001 |
| HSPA13 | PRAD | T cells CD8 | -0.31 | <0.001 |
| HSPA13 | KICH | NK cells activated | -0.35 | 0.035 |
| HSPA13 | THYM | T cells CD8 | -0.35 | <0.001 |
| HSPA13 | KICH | Macrophages M2 | -0.37 | 0.027 |
| HSPA13 | UCS | B cells memory | -0.43 | 0.007 |
| HSPA13 | ACC | Mast cells resting | -0.47 | 0.002 |
| HSPA13 | UVM | Monocytes | -0.62 | <0.001 |
| HSPA12B | KICH | Macrophages M2 | 0.43 | 0.010 |
| HSPA12B | ACC | B cells naive | 0.41 | 0.009 |
| HSPA12B | UCS | Macrophages M2 | 0.39 | 0.015 |
| HSPA12B | ACC | Macrophages M1 | 0.37 | 0.018 |
| HSPA12B | CHOL | Plasma cells | 0.36 | 0.040 |
| HSPA12B | UVM | Monocytes | 0.36 | 0.041 |
| HSPA12B | UVM | NK cells resting | 0.35 | 0.046 |
| HSPA12B | ACC | B cells naive | 0.34 | <0.001 |
| HSPA12B | ACC | Macrophages M0 | 0.33 | 0.037 |
| HSPA12B | KIRP | B cells naive | 0.33 | <0.001 |
| HSPA12B | STAD | Monocytes | 0.32 | <0.001 |
| HSPA12B | PAAD | B cells naive | 0.31 | <0.001 |
| HSPA12B | DLBC | Dendritic cells resting | -0.32 | 0.026 |
| HSPA12B | UCS | B cells memory | -0.32 | 0.047 |
| HSPA12B | ACC | Dendritic cells activated | -0.33 | <0.001 |
| HSPA12B | ACC | T cells follicular helper | -0.34 | <0.001 |
| HSPA12B | ESCA | Dendritic cells activated | -0.35 | <0.001 |
| HSPA12B | TGCT | B cells naive | -0.36 | <0.001 |
| HSPA12B | THCA | Dendritic cells activated | -0.36 | <0.001 |
| HSPA12B | MESO | NK cells activated | -0.37 | 0.001 |
| HSPA12B | UVM | T cells follicular helper | -0.37 | 0.033 |
| HSPA12B | UCS | Mast cells activated | -0.43 | 0.007 |
| HSPA12B | ACC | Dendritic cells activated | -0.49 | 0.001 |
| HSPA12A | ACC | Dendritic cells activated | 0.47 | 0.003 |
| HSPA12A | KICH | Monocytes | 0.4 | 0.017 |
| HSPA12A | KICH | Monocytes | 0.4 | 0.017 |
| HSPA12A | CHOL | Macrophages M2 | 0.38 | 0.030 |
| HSPA12A | CHOL | Macrophages M2 | 0.38 | 0.030 |
| HSPA12A | UVM | Mast cells resting | 0.38 | 0.030 |
| HSPA12A | CHOL | Dendritic cells resting | 0.37 | 0.036 |
| HSPA12A | CHOL | Dendritic cells resting | 0.37 | 0.036 |
| HSPA12A | THYM | T cells CD4 memory resting | 0.35 | <0.001 |
| HSPA12A | UCS | B cells naive | 0.35 | 0.031 |
| HSPA12A | HNSC | T cells CD4 memory resting | 0.34 | <0.001 |
| HSPA12A | HNSC | T cells CD4 memory resting | 0.34 | <0.001 |
| HSPA12A | HNSC | T cells CD4 memory resting | 0.34 | <0.001 |
| HSPA12A | ACC | Eosinophils | 0.32 | 0.045 |
| HSPA12A | DLBC | Macrophages M0 | 0.31 | 0.032 |
| HSPA12A | DLBC | Macrophages M0 | 0.31 | 0.032 |
| HSPA12A | DLBC | Macrophages M0 | 0.31 | 0.032 |
| HSPA12A | MESO | Monocytes | 0.3 | 0.006 |
| HSPA12A | MESO | Monocytes | 0.3 | 0.006 |
| HSPA12A | MESO | Monocytes | 0.3 | 0.006 |
| HSPA12A | DLBC | Macrophages M2 | -0.31 | 0.030 |
| HSPA12A | DLBC | Macrophages M2 | -0.31 | 0.030 |
| HSPA12A | DLBC | Macrophages M2 | -0.31 | 0.030 |
| HSPA12A | ACC | Macrophages M1 | -0.33 | 0.040 |
| HSPA12A | ACC | T cells CD8 | -0.33 | 0.037 |
| HSPA12A | TGCT | B cells naive | -0.35 | <0.001 |
| HSPA12A | TGCT | B cells naive | -0.35 | <0.001 |
| HSPA12A | UCS | Mast cells resting | -0.35 | 0.031 |
| HSPA12A | HNSC | T cells CD8 | -0.36 | <0.001 |
| HSPA12A | MESO | T cells CD4 memory activated | -0.36 | 0.001 |
| HSPA12A | PAAD | B cells memory | -0.36 | <0.001 |
| HSPA12A | HNSC | T cells CD8 | -0.36 | <0.001 |
| HSPA12A | MESO | T cells CD4 memory activated | -0.36 | 0.001 |
| HSPA12A | PAAD | B cells memory | -0.36 | <0.001 |
| HSPA12A | HNSC | T cells CD8 | -0.36 | <0.001 |
| HSPA12A | MESO | T cells CD4 memory activated | -0.36 | 0.001 |
| HSPA12A | PAAD | B cells memory | -0.36 | <0.001 |
| HSPA12A | UCS | B cells memory | -0.38 | 0.018 |
| HSPA12A | CHOL | T cells follicular helper | -0.4 | 0.022 |
| HSPA12A | CHOL | T cells follicular helper | -0.4 | 0.022 |
| HSPA12A | ACC | T cells CD4 memory activated | -0.46 | 0.003 |
| HSPA12A | UVM | B cells memory | -0.46 | 0.008 |
| HSPA12A | CHOL | T cells CD8 | -0.51 | 0.003 |
| HSPA12A | CHOL | T cells CD8 | -0.51 | 0.003 |
| HSPA12A | KICH | Macrophages M0 | -0.54 | 0.001 |
| HSPA12A | KICH | Macrophages M0 | -0.54 | 0.001 |
| HSPA12A | KICH | Macrophages M0 | -0.54 | 0.001 |
| HSP90B1 | UVM | T cells follicular helper | 0.5 | 0.003 |
| HSP90B1 | CHOL | Macrophages M0 | 0.46 | 0.007 |
| HSP90B1 | ACC | Macrophages M0 | 0.44 | 0.004 |
| HSP90B1 | UVM | T cells CD4 memory activated | 0.4 | 0.021 |
| HSP90B1 | KICH | T cells CD4 memory resting | 0.37 | 0.026 |
| HSP90B1 | CHOL | T cells regulatory (Tregs) | 0.37 | 0.032 |
| HSP90B1 | CHOL | Neutrophils | 0.37 | 0.033 |
| HSP90B1 | DLBC | T cells CD4 memory activated | 0.36 | 0.013 |
| HSP90B1 | DLBC | Dendritic cells activated | 0.35 | 0.016 |
| HSP90B1 | TGCT | Dendritic cells activated | 0.34 | <0.001 |
| HSP90B1 | TGCT | T cells CD4 memory resting | -0.3 | <0.001 |
| HSP90B1 | THCA | Dendritic cells resting | -0.3 | <0.001 |
| HSP90B1 | THYM | T cells follicular helper | -0.33 | <0.001 |
| HSP90B1 | PCPG | NK cells activated | -0.35 | 0.003 |
| HSP90B1 | THCA | Dendritic cells activated | -0.36 | <0.001 |
| HSP90B1 | TGCT | B cells naive | -0.45 | <0.001 |
| HSP90B1 | UVM | Plasma cells | -0.48 | 0.004 |
| HSP90B1 | UVM | Monocytes | -0.48 | 0.004 |
| HSP90AB1 | TGCT | B cells naive | 0.44 | <0.001 |
| HSP90AB1 | UCS | NK cells resting | 0.41 | 0.010 |
| HSP90AB1 | CHOL | Plasma cells | 0.4 | 0.020 |
| HSP90AB1 | CHOL | Neutrophils | 0.4 | 0.023 |
| HSP90AB1 | LAML | Eosinophils | 0.39 | <0.001 |
| HSP90AB1 | KICH | T cells CD4 memory resting | 0.35 | 0.040 |
| HSP90AB1 | THYM | NK cells activated | 0.34 | <0.001 |
| HSP90AB1 | THYM | Mast cells resting | 0.31 | 0.001 |
| HSP90AB1 | GBM | T cells gamma delta | -0.3 | <0.001 |
| HSP90AB1 | ACC | T cells follicular helper | -0.33 | 0.037 |
| HSP90AB1 | UVM | B cells memory | -0.42 | 0.015 |
| HSP90AB1 | UCS | T cells gamma delta | -0.42 | 0.008 |
| HSP90AB1 | DLBC | T cells regulatory (Tregs) | -0.46 | 0.001 |
| HSP90AB1 | KICH | Macrophages M2 | -0.48 | 0.004 |
| HSP90AA1 | TGCT | B cells naive | 0.46 | <0.001 |
| HSP90AA1 | UVM | T cells follicular helper | 0.44 | 0.011 |
| HSP90AA1 | UCS | NK cells resting | 0.43 | 0.006 |
| HSP90AA1 | CHOL | Mast cells activated | 0.43 | 0.013 |
| HSP90AA1 | UVM | T cells CD4 memory activated | 0.4 | 0.022 |
| HSP90AA1 | CHOL | Neutrophils | 0.4 | 0.020 |
| HSP90AA1 | KICH | B cells naive | 0.39 | 0.019 |
| HSP90AA1 | UCS | Macrophages M0 | 0.39 | 0.014 |
| HSP90AA1 | KICH | Macrophages M1 | 0.37 | 0.026 |
| HSP90AA1 | UCS | Mast cells activated | 0.36 | 0.026 |
| HSP90AA1 | CHOL | Macrophages M0 | 0.35 | 0.048 |
| HSP90AA1 | KICH | T cells CD4 memory resting | 0.34 | 0.046 |
| HSP90AA1 | DLBC | T cells CD4 memory activated | 0.34 | 0.020 |
| HSP90AA1 | DLBC | NK cells activated | 0.33 | 0.020 |
| HSP90AA1 | LAML | Eosinophils | 0.32 | <0.001 |
| HSP90AA1 | LAML | T cells CD8 | -0.3 | <0.001 |
| HSP90AA1 | STAD | Monocytes | -0.31 | <0.001 |
| HSP90AA1 | ACC | B cells memory | -0.38 | 0.002 |
| HSP90AA1 | DLBC | Macrophages M1 | -0.39 | 0.006 |
| HSP90AA1 | UVM | Plasma cells | -0.46 | 0.007 |
| HSP90AA1 | CHOL | NK cells activated | -0.55 | 0.020 |
| HSP90AA1 | UVM | Monocytes | -0.61 | <0.001 |
| HSPD1 | CHOL | B cells memory | -0.35 | 0.046 |
| HSPD1 | TGCT | B cells naive | 0.3 | <0.001 |
| HSPD1 | PCPG | B cells naive | 0.33 | 0.005 |
| HSPD1 | ACC | Dendritic cells activated | 0.44 | 0.005 |
| HSPD1 | ACC | Eosinophils | 0.63 | <0.001 |
| HSPD1 | LAML | Eosinophils | 0.31 | <0.001 |
| HSPD1 | KICH | Macrophages M0 | -0.33 | 0.047 |
| HSPD1 | CHOL | Macrophages M1 | 0.36 | 0.041 |
| HSPD1 | KICH | Macrophages M2 | -0.5 | 0.002 |
| HSPD1 | UCS | Mast cells resting | -0.38 | 0.016 |
| HSPD1 | CHOL | Mast cells resting | -0.43 | 0.014 |
| HSPD1 | ACC | Mast cells resting | 0.39 | 0.013 |
| HSPD1 | UVM | Monocytes | -0.56 | 0.001 |
| HSPD1 | STAD | Monocytes | -0.32 | <0.001 |
| HSPD1 | CHOL | Neutrophils | 0.45 | 0.009 |
| HSPD1 | KICH | T cells CD4 memory resting | 0.59 | <0.001 |
| HSPD1 | CHOL | T cells CD8 | -0.38 | 0.032 |
| HSPD1 | ACC | T cells CD8 | -0.59 | <0.001 |
| HSPD1 | UVM | T cells follicular helper | 0.41 | 0.019 |
| HSPD1 | STAD | T cells follicular helper | 0.3 | <0.001 |
| HSPD1 | ACC | T cells gamma delta | -0.4 | 0.011 |
| HSPD1 | DLBC | T cells regulatory (T regs) | -0.33 | 0.021 |
| HSPD1 | ACC | T cells regulatory (T regs) | -0.32 | 0.047 |

| **Table S5 The relationships between heat shock proteins expressions and copy number variations in pan-cancer** | | | | | |
| --- | --- | --- | --- | --- | --- |
| **Gene name** | **CNVCat** | **N** | **summarise** | ***P* value** | **CancerType** |
| HSPA7 | DEL | 2 | 9.116(8.612-9.62) | < 0.001 | BLCA |
|  | GAIN | 100 | 9.186(8.103-10.59) |  |  |
|  | No Change | 306 | 8.248(7.51-9.147) |  |  |
|  | DEL | 19 | 8.124(7.768-8.897) | < 0.001 | BRCA |
|  | GAIN | 156 | 8.553(7.705-9.555) |  |  |
|  | No Change | 915 | 8.028(7.18-8.932) |  |  |
|  | GAIN | 71 | 9.008(7.974-10.058) | 0.021679 | LUAD |
|  | No Change | 453 | 8.558(7.693-9.502) |  |  |
|  | DEL | 1 | 7.672(7.672-7.672) | 0.015 | LUSC |
|  | GAIN | 57 | 9.292(8.455-10.394) |  |  |
|  | No Change | 442 | 8.815(8.062-9.587) |  |  |
|  | GAIN | 9 | 8.922(8.234-10.934) | 0.005417 | PAAD |
|  | No Change | 168 | 7.827(7.053-8.625) |  |  |
|  | GAIN | 3 | 8.29(7.899-8.826) | 0.037972 | THCA |
|  | No Change | 504 | 6.794(5.901-7.604) |  |  |
| HSPA1B | DEL | 11 | 13.117(12.543-13.416) | 0.01 | BLCA |
|  | GAIN | 41 | 13.512(12.394-13.906) |  |  |
|  | No Change | 356 | 12.752(12.119-13.453) |  |  |
|  | DEL | 29 | 11.974(11.284-12.889) | < 0.001 | BRCA |
|  | GAIN | 79 | 13.105(12.342-13.503) |  |  |
|  | No Change | 982 | 12.531(12.001-13.075) |  |  |
|  | DEL | 7 | 12.192(10.823-12.672) | < 0.001 | ESCA |
|  | GAIN | 18 | 13.512(13.097-14.047) |  |  |
|  | No Change | 136 | 12.441(11.876-13.152) |  |  |
|  | DEL | 16 | 11.432(10.902-12.141) | < 0.001 | HNSC |
|  | GAIN | 12 | 12.996(12.475-13.488) |  |  |
|  | No Change | 468 | 12.41(11.838-13.024) |  |  |
|  | DEL | 23 | 12.47(11.725-12.859) | 0.048 | OV |
|  | GAIN | 71 | 12.733(12.273-13.244) |  |  |
|  | No Change | 283 | 12.53(11.978-13.053) |  |  |
|  | DEL | 9 | 12.22(11.488-12.48) | 0.008 | SKCM |
|  | GAIN | 31 | 11.996(11.595-12.448) |  |  |
|  | No Change | 430 | 11.502(10.95-12.149) |  |  |
|  | DEL | 3 | 10.705(10.514-11.863) | 0.033 | UCEC |
|  | GAIN | 19 | 12.328(11.929-13.297) |  |  |
|  | No Change | 518 | 11.825(11.133-12.567) |  |  |
| HSPA4L | DEL | 17 | 7.313(6.57-7.895) | 0.029 | BLCA |
|  | GAIN | 17 | 8.741(6.895-9.783) |  |  |
|  | No Change | 374 | 8.574(7.26-9.839) |  |  |
|  | DEL | 27 | 8.093(7.588-8.927) | < 0.001 | BRCA |
|  | GAIN | 41 | 10.197(8.683-11.126) |  |  |
|  | No Change | 1022 | 9.172(8.19-10.123) |  |  |
|  | DEL | 14 | 8.16(6.872-8.95) | 0.022 | COAD |
|  | GAIN | 3 | 3.907(3.357-5.068) |  |  |
|  | No Change | 445 | 8.558(7.011-9.755) |  |  |
|  | DEL | 8 | 9.935(8.946-10.414) | 0.003 | KIRC |
|  | GAIN | 2 | 11.65(11.317-11.982) |  |  |
|  | No Change | 520 | 11.168(10.355-11.643) |  |  |
|  | DEL | 20 | 8.515(7.475-8.879) | 0.04 | LUAD |
|  | GAIN | 8 | 9.654(9.103-9.943) |  |  |
|  | No Change | 496 | 8.628(7.864-9.527) |  |  |
|  | DEL | 32 | 10.089(8.893-11.237) | 0.046 | LUSC |
|  | GAIN | 8 | 11.714(10.299-11.999) |  |  |
|  | No Change | 460 | 10.512(9.679-11.263) |  |  |
|  | DEL | 4 | 6.553(6.394-7.212) | 0.032604 | PAAD |
|  | No Change | 173 | 8.615(7.883-9.386) |  |  |
|  | DEL | 16 | 9.187(7.434-9.728) | 0.013 | STAD |
|  | GAIN | 8 | 9.083(8.263-9.454) |  |  |
|  | No Change | 349 | 9.895(8.849-10.82) |  |  |
| HSPA4 | DEL | 11 | 12.606(12.011-12.749) | 0.04 | BLCA |
|  | GAIN | 13 | 13.311(12.599-14.029) |  |  |
|  | No Change | 384 | 12.785(12.348-13.213) |  |  |
|  | DEL | 34 | 12.906(12.542-13.312) | < 0.001 | BRCA |
|  | GAIN | 29 | 13.933(13.387-14.27) |  |  |
|  | No Change | 1027 | 13.426(12.916-13.866) |  |  |
|  | DEL | 8 | 12.398(11.917-12.712) | 0.009 | CESC |
|  | GAIN | 4 | 13.126(12.808-13.623) |  |  |
|  | No Change | 282 | 12.913(12.502-13.34) |  |  |
|  | DEL | 15 | 11.859(11.483-12.253) | 0.046 | COAD |
|  | GAIN | 2 | 11.369(11.082-11.656) |  |  |
|  | No Change | 445 | 12.388(11.524-12.913) |  |  |
|  | DEL | 15 | 12.403(12.152-12.94) | < 0.001 | HNSC |
|  | GAIN | 6 | 14.161(13.682-14.456) |  |  |
|  | No Change | 475 | 13.182(12.763-13.557) |  |  |
|  | DEL | 6 | 12.155(11.963-12.281) | < 0.001 | KIRC |
|  | GAIN | 110 | 12.833(12.523-13.19) |  |  |
|  | No Change | 414 | 12.594(12.141-12.98) |  |  |
|  | DEL | 2 | 11.732(11.646-11.818) | 0.011 | KIRP |
|  | GAIN | 5 | 13.122(13.049-14.137) |  |  |
|  | No Change | 280 | 12.646(12.112-13.057) |  |  |
|  | DEL | 7 | 10.926(10.754-11.136) | 0.00523 | LAML |
|  | No Change | 125 | 11.723(11.3-11.914) |  |  |
|  | DEL | 11 | 12.214(11.586-12.51) | < 0.001 | LGG |
|  | GAIN | 1 | 13.793(13.793-13.793) |  |  |
|  | No Change | 516 | 12.69(12.39-13.057) |  |  |
|  | DEL | 7 | 11.343(11.213-11.616) | < 0.001 | LIHC |
|  | GAIN | 9 | 13.161(12.934-13.289) |  |  |
|  | No Change | 356 | 12.426(12.071-12.883) |  |  |
|  | DEL | 35 | 12.169(11.514-12.94) | 0.037 | LUAD |
|  | GAIN | 4 | 11.271(11.085-11.916) |  |  |
|  | No Change | 485 | 12.433(11.951-13.038) |  |  |
|  | DEL | 20 | 12.826(12.336-13.109) | 0.038 | LUSC |
|  | GAIN | 5 | 13.786(13.703-14.055) |  |  |
|  | No Change | 475 | 12.871(12.398-13.246) |  |  |
|  | DEL | 36 | 12.227(11.927-12.766) | < 0.001 | OV |
|  | GAIN | 42 | 13.009(12.629-13.298) |  |  |
|  | No Change | 299 | 12.718(12.179-13.187) |  |  |
|  | DEL | 4 | 11.917(11.584-12.025) | 0.002876 | PCPG |
|  | No Change | 164 | 12.906(12.459-13.236) |  |  |
|  | DEL | 19 | 12.108(11.513-12.437) | 0.005 | PRAD |
|  | GAIN | 5 | 12.339(12.246-13.347) |  |  |
|  | No Change | 472 | 12.469(12.1-12.828) |  |  |
|  | DEL | 14 | 12.308(11.65-12.458) | 0.015 | SARC |
|  | GAIN | 9 | 12.865(12.593-13.196) |  |  |
|  | No Change | 239 | 12.507(12.093-12.876) |  |  |
|  | DEL | 28 | 12.782(12.308-13.115) | < 0.001 | SKCM |
|  | GAIN | 7 | 13.617(12.998-14.148) |  |  |
|  | No Change | 435 | 13.379(12.834-13.908) |  |  |
|  | DEL | 20 | 12.49(12.201-12.772) | 0.025 | STAD |
|  | GAIN | 4 | 13.108(13.087-13.253) |  |  |
|  | No Change | 349 | 12.848(12.345-13.2) |  |  |
|  | DEL | 8 | 11.792(11.217-12.468) | 0.031 | UCEC |
|  | GAIN | 6 | 13.225(12.695-13.383) |  |  |
|  | No Change | 526 | 12.173(11.671-12.902) |  |  |
|  | DEL | 1 | 12.872(12.872-12.872) | 0.008 | UCS |
|  | GAIN | 5 | 13.851(13.827-14.056) |  |  |
|  | No Change | 49 | 13.136(12.854-13.486) |  |  |
| HSPA9 | DEL | 20 | 13.876(13.445-14.216) | < 0.001 | BRCA |
|  | GAIN | 46 | 14.78(14.374-15.392) |  |  |
|  | No Change | 1024 | 14.217(13.78-14.684) |  |  |
|  | DEL | 4 | 13.146(12.784-13.497) | 0.008 | CESC |
|  | GAIN | 8 | 14.298(14.05-14.587) |  |  |
|  | No Change | 282 | 13.746(13.321-14.144) |  |  |
|  | DEL | 6 | 12.974(12.589-13.606) | 0.039 | COAD |
|  | GAIN | 4 | 12.486(12.322-12.648) |  |  |
|  | No Change | 452 | 13.544(12.785-14.199) |  |  |
|  | DEL | 6 | 13.382(13.256-13.511) | 0.011 | HNSC |
|  | GAIN | 5 | 14.341(14.188-14.431) |  |  |
|  | No Change | 485 | 13.691(13.278-14.091) |  |  |
|  | GAIN | 113 | 14.477(14.073-14.883) | < 0.001 | KIRC |
|  | No Change | 417 | 14.201(13.685-14.632) |  |  |
|  | DEL | 2 | 12.75(12.648-12.852) | 0.015 | KIRP |
|  | GAIN | 5 | 14.473(14.188-15.446) |  |  |
|  | No Change | 280 | 13.82(13.389-14.358) |  |  |
|  | DEL | 7 | 11.952(11.574-12.151) | < 0.001 | LAML |
|  | No Change | 125 | 12.595(12.33-12.903) |  |  |
|  | DEL | 10 | 13.639(13.305-13.965) | 0.002 | LGG |
|  | GAIN | 1 | 14.644(14.644-14.644) |  |  |
|  | No Change | 517 | 14.142(13.842-14.463) |  |  |
|  | DEL | 4 | 13.63(13.359-13.745) | 0.044 | LIHC |
|  | GAIN | 18 | 14.573(13.939-14.875) |  |  |
|  | No Change | 350 | 14.206(13.727-14.617) |  |  |
|  | DEL | 12 | 13.37(13.197-13.694) | < 0.001 | LUSC |
|  | GAIN | 12 | 14.562(14.26-14.87) |  |  |
|  | No Change | 476 | 13.655(13.212-14.097) |  |  |
|  | DEL | 24 | 13.111(12.832-13.617) | < 0.001 | OV |
|  | GAIN | 55 | 14.126(13.695-14.519) |  |  |
|  | No Change | 298 | 13.618(13.041-14.112) |  |  |
|  | GAIN | 4 | 14.408(13.914-14.562) | 0.037738 | PAAD |
|  | No Change | 173 | 13.182(12.811-13.531) |  |  |
|  | DEL | 5 | 13.244(12.987-13.283) | 0.002272 | PCPG |
|  | No Change | 163 | 14.08(13.736-14.421) |  |  |
|  | DEL | 10 | 13.543(13.249-13.683) | 0.025 | PRAD |
|  | GAIN | 7 | 14.326(13.564-14.526) |  |  |
|  | No Change | 479 | 13.89(13.532-14.282) |  |  |
|  | DEL | 37 | 13.936(13.484-14.199) | 0.001 | SKCM |
|  | GAIN | 11 | 14.68(14.226-15.138) |  |  |
|  | No Change | 422 | 14.264(13.743-14.768) |  |  |
|  | DEL | 11 | 13.818(13.513-13.968) | 0.025 | STAD |
|  | GAIN | 11 | 14.649(13.837-14.955) |  |  |
|  | No Change | 351 | 13.724(13.214-14.221) |  |  |
|  | DEL | 4 | 13.084(12.669-13.56) | < 0.001 | UCEC |
|  | GAIN | 20 | 13.309(13.02-14.356) |  |  |
|  | No Change | 516 | 12.639(12.125-13.335) |  |  |
|  | GAIN | 5 | 14.722(14.075-14.792) | 0.036322 | UCS |
|  | No Change | 50 | 13.732(13.381-14.049) |  | UCS |
| HSPA1L | DEL | 11 | 6.248(5.326-6.895) | 0.046 | BLCA |
|  | GAIN | 41 | 6.7(6.322-7.129) |  |  |
|  | No Change | 356 | 6.331(5.781-6.883) |  |  |
|  | DEL | 29 | 7.16(6.426-7.707) | 0.004 | BRCA |
|  | GAIN | 79 | 7.781(7.361-8.25) |  |  |
|  | No Change | 982 | 7.658(7.14-8.124) |  |  |
|  | DEL | 7 | 5.087(4.779-5.773) | 0.035 | COAD |
|  | GAIN | 12 | 6.303(5.882-6.932) |  |  |
|  | No Change | 443 | 5.585(5-6.257) |  |  |
|  | DEL | 7 | 6.459(5.82-7.822) | 0.022 | ESCA |
|  | GAIN | 18 | 7.515(6.947-7.787) |  |  |
|  | No Change | 136 | 6.801(6.455-7.267) |  |  |
|  | DEL | 3 | 6.044(5.913-6.276) | 0.019502 | KIRP |
|  | No Change | 284 | 7.175(6.672-7.687) |  |  |
|  | DEL | 23 | 7.409(6.721-7.829) | 0.047 | OV |
|  | GAIN | 71 | 7.7(7.15-8.152) |  |  |
|  | No Change | 283 | 7.358(6.807-8.033) |  |  |
|  | DEL | 9 | 6.15(5.883-6.508) | 0.032 | STAD |
|  | GAIN | 29 | 6.907(6.629-7.443) |  |  |
|  | No Change | 335 | 6.82(6.322-7.257) |  |  |
| HSPA1A | DEL | 29 | 12.288(11.28-12.908) | 0.009 | BRCA |
|  | GAIN | 79 | 12.89(12.124-13.476) |  |  |
|  | No Change | 982 | 12.624(12.003-13.265) |  |  |
|  | DEL | 2 | 12.277(11.876-12.677) | 0.017 | CESC |
|  | GAIN | 23 | 13.377(12.971-14.015) |  |  |
|  | No Change | 269 | 12.79(12.181-13.443) |  |  |
|  | DEL | 7 | 10.932(10.734-12.414) | 0.002 | ESCA |
|  | GAIN | 18 | 13.436(12.678-14.478) |  |  |
|  | No Change | 136 | 12.491(11.285-13.252) |  |  |
|  | DEL | 16 | 11.577(10.944-12.23) | 0.004 | HNSC |
|  | GAIN | 12 | 12.87(12.22-13.707) |  |  |
|  | No Change | 468 | 12.311(11.625-13.08) |  |  |
|  | DEL | 3 | 8.948(8.763-9.879) | 0.030975 | KIRP |
|  | No Change | 284 | 11.292(10.542-12.053) |  |  |
|  | DEL | 3 | 13.241(12.911-13.488) | 0.012 | LIHC |
|  | GAIN | 22 | 12.987(12.205-13.439) |  |  |
|  | No Change | 347 | 12.273(11.494-13.063) |  |  |
|  | DEL | 23 | 11.534(9.235-12.748) | 0.046 | OV |
|  | GAIN | 71 | 12.383(11.084-13.199) |  |  |
|  | No Change | 283 | 11.913(10.861-12.64) |  |  |
|  | DEL | 9 | 12.157(11.75-12.425) | 0.002 | SKCM |
|  | GAIN | 31 | 12.258(11.864-12.847) |  |  |
|  | No Change | 430 | 11.755(11.139-12.355) |  |  |
|  | DEL | 9 | 11.02(10.69-11.526) | 0.037 | STAD |
|  | GAIN | 29 | 12.183(11.615-13.181) |  |  |
|  | No Change | 335 | 11.418(10.257-12.42) |  |  |
| HSP90AB1 | DEL | 5 | 16.694(16.003-16.764) | < 0.001 | BLCA |
|  | GAIN | 36 | 16.94(16.148-17.392) |  |  |
|  | No Change | 367 | 16.006(15.55-16.518) |  |  |
|  | DEL | 25 | 16.003(15.677-16.781) | < 0.001 | BRCA |
|  | GAIN | 70 | 17.149(16.617-17.679) |  |  |
|  | No Change | 995 | 16.527(16.092-17.019) |  |  |
|  | DEL | 4 | 15.607(15.227-15.918) | < 0.001 | CESC |
|  | GAIN | 11 | 16.866(16.472-17.291) |  |  |
|  | No Change | 279 | 15.902(15.405-16.444) |  |  |
|  | DEL | 1 | 13.375(13.375-13.375) | 0.004 | COAD |
|  | GAIN | 13 | 16.367(16.191-16.671) |  |  |
|  | No Change | 448 | 15.71(14.903-16.296) |  |  |
|  | DEL | 3 | 16.339(16.223-16.523) | < 0.001 | ESCA |
|  | GAIN | 30 | 17.57(17.006-18.167) |  |  |
|  | No Change | 128 | 16.813(16.477-17.086) |  |  |
|  | DEL | 6 | 15.795(15.11-15.957) | 0.003 | HNSC |
|  | GAIN | 15 | 17.051(16.336-17.401) |  |  |
|  | No Change | 475 | 16.207(15.839-16.647) |  |  |
|  | DEL | 3 | 14.327(13.991-14.665) | 0.004 | KIRC |
|  | GAIN | 2 | 16.923(16.749-17.096) |  |  |
|  | No Change | 525 | 15.863(15.45-16.231) |  |  |
|  | DEL | 1 | 15.927(15.927-15.927) | 0.043 | KIRP |
|  | GAIN | 2 | 17.623(17.454-17.791) |  |  |
|  | No Change | 284 | 15.451(15.003-15.943) |  |  |
|  | DEL | 2 | 15.113(14.965-15.26) | 0.024 | LGG |
|  | GAIN | 3 | 16.587(16.27-16.676) |  |  |
|  | No Change | 523 | 15.775(15.422-16.13) |  |  |
|  | DEL | 17 | 15.674(15.346-16.009) | < 0.001 | LUSC |
|  | GAIN | 32 | 16.869(16.357-17.378) |  |  |
|  | No Change | 451 | 16.283(15.823-16.713) |  |  |
|  | DEL | 21 | 16.284(15.321-16.799) | < 0.001 | OV |
|  | GAIN | 50 | 16.808(16.389-17.181) |  |  |
|  | No Change | 306 | 16.389(15.93-16.878) |  |  |
|  | DEL | 1 | 14.448(14.448-14.448) | 0.039 | READ |
|  | GAIN | 12 | 15.934(15.566-17.068) |  |  |
|  | No Change | 152 | 15.812(14.837-16.511) |  |  |
|  | DEL | 6 | 15.028(14.879-15.131) | < 0.001 | SARC |
|  | GAIN | 19 | 16.011(15.419-16.958) |  |  |
|  | No Change | 237 | 15.418(14.965-15.901) |  |  |
|  | DEL | 12 | 17.027(16.428-17.469) | 0.029 | SKCM |
|  | GAIN | 22 | 17.215(16.886-17.749) |  |  |
|  | No Change | 436 | 16.819(16.394-17.368) |  |  |
|  | DEL | 2 | 16.4(16.031-16.768) | < 0.001 | STAD |
|  | GAIN | 46 | 17.057(16.612-17.592) |  |  |
|  | No Change | 325 | 16.184(15.797-16.644) |  |  |
|  | DEL | 4 | 15.22(15.089-15.392) | < 0.001 | UCEC |
|  | GAIN | 22 | 16.129(15.789-16.775) |  |  |
|  | No Change | 514 | 15.288(14.703-15.923) |  |  |
| HSPA5 | DEL | 6 | 14.077(13.97-14.715) | 0.027 | BLCA |
|  | GAIN | 14 | 15.021(14.943-15.839) |  |  |
|  | No Change | 388 | 14.907(14.388-15.452) |  |  |
|  | DEL | 24 | 15.473(15.216-15.839) | < 0.001 | BRCA |
|  | GAIN | 61 | 15.908(15.41-16.327) |  |  |
|  | No Change | 1005 | 15.398(14.949-15.838) |  |  |
|  | DEL | 7 | 15.764(15.47-15.82) | 0.023 | HNSC |
|  | GAIN | 19 | 15.783(15.538-16.106) |  |  |
|  | No Change | 470 | 15.441(14.961-15.849) |  |  |
|  | DEL | 35 | 14.894(14.024-15.177) | 0.017 | OV |
|  | GAIN | 31 | 15.189(14.871-15.597) |  |  |
|  | No Change | 311 | 14.975(14.383-15.442) |  |  |
| HSPA14 | DEL | 6 | 9.395(8.886-9.736) | < 0.001 | BLCA |
|  | GAIN | 32 | 11.093(10.653-11.604) |  |  |
|  | No Change | 370 | 10.424(9.904-10.864) |  |  |
|  | DEL | 8 | 10.562(10.287-10.714) | < 0.001 | BRCA |
|  | GAIN | 79 | 11.775(11.177-12.302) |  |  |
|  | No Change | 1003 | 10.77(10.373-11.209) |  |  |
|  | DEL | 9 | 9.78(9.255-10.015) | < 0.001 | HNSC |
|  | GAIN | 10 | 11.205(10.603-11.316) |  |  |
|  | No Change | 477 | 10.373(9.925-10.807) |  |  |
|  | DEL | 2 | 9.575(9.411-9.74) | < 0.001 | LGG |
|  | GAIN | 10 | 10.945(10.67-11.437) |  |  |
|  | No Change | 516 | 10.258(9.967-10.557) |  |  |
|  | DEL | 15 | 9.658(9.019-10.147) | 0.009 | LUAD |
|  | GAIN | 19 | 10.574(9.79-11.138) |  |  |
|  | No Change | 490 | 10.223(9.675-10.791) |  |  |
|  | DEL | 11 | 9.781(9.639-10.255) | < 0.001 | LUSC |
|  | GAIN | 12 | 11.397(11.079-11.696) |  |  |
|  | No Change | 477 | 10.672(10.249-11.157) |  |  |
|  | DEL | 15 | 10.367(10.005-10.468) | < 0.001 | OV |
|  | GAIN | 48 | 11.303(10.672-11.682) |  |  |
|  | No Change | 314 | 10.739(10.149-11.222) |  |  |
|  | DEL | 3 | 9.484(8.947-9.761) | 0.019 | PAAD |
|  | GAIN | 3 | 10.738(10.715-10.884) |  |  |
|  | No Change | 171 | 9.864(9.429-10.197) |  |  |
|  | DEL | 9 | 9.484(8.931-9.74) | 0.003 | PRAD |
|  | GAIN | 4 | 10.441(10.28-10.578) |  |  |
|  | No Change | 483 | 10.047(9.653-10.44) |  |  |
|  | DEL | 7 | 9.763(9.354-9.938) | < 0.001 | SARC |
|  | GAIN | 9 | 10.996(10.914-11.468) |  |  |
|  | No Change | 246 | 9.943(9.513-10.416) |  |  |
|  | DEL | 7 | 10.799(10.329-11.012) | 0.002 | STAD |
|  | GAIN | 18 | 11.612(11.116-12.08) |  |  |
|  | No Change | 348 | 10.911(10.444-11.336) |  |  |
|  | DEL | 5 | 8.683(8.165-9.278) | 0.005 | UCEC |
|  | GAIN | 17 | 10.399(9.52-10.644) |  |  |
|  | No Change | 518 | 9.405(8.792-10.12) |  |  |
| HSPA12A | GAIN | 4 | 13.078(12.657-13.284) | 0.003688 | ACC |
|  | No Change | 73 | 11.049(10.224-11.644) |  |  |
|  | DEL | 76 | 8.687(8.185-9.419) | < 0.001 | BRCA |
|  | GAIN | 32 | 9.39(8.369-9.809) |  |  |
|  | No Change | 982 | 9.271(8.574-9.949) |  |  |
|  | DEL | 13 | 8.18(7.693-9.02) | 0.039 | CESC |
|  | GAIN | 4 | 8.355(7.09-10.225) |  |  |
|  | No Change | 277 | 7.2(6.19-8.313) |  |  |
|  | DEL | 2 | 7.472(6.966-7.978) | 0.033 | DLBC |
|  | GAIN | 3 | 4.954(4.906-4.954) |  |  |
|  | No Change | 43 | 5.728(5.129-6.486) |  |  |
|  | DEL | 12 | 6.548(5.03-8.612) | 0.011 | HNSC |
|  | GAIN | 7 | 9.028(7.476-9.207) |  |  |
|  | No Change | 477 | 8.7(7.672-9.683) |  |  |
|  | DEL | 5 | 10.338(10.093-10.568) | 0.045 | KIRC |
|  | GAIN | 1 | 12.448(12.448-12.448) |  |  |
|  | No Change | 524 | 11.263(10.509-11.795) |  |  |
|  | DEL | 37 | 10.438(9.875-11.014) | < 0.001 | LGG |
|  | GAIN | 2 | 12.121(11.638-12.604) |  |  |
|  | No Change | 489 | 11.094(10.495-11.835) |  |  |
|  | DEL | 10 | 6.52(5.653-6.888) | 0.002 | LUAD |
|  | GAIN | 14 | 8.169(7.677-8.654) |  |  |
|  | No Change | 500 | 7.524(6.697-8.172) |  |  |
|  | DEL | 5 | 8.861(8.842-10.551) | 0.025 | SARC |
|  | GAIN | 20 | 10.012(9.63-10.809) |  |  |
|  | No Change | 237 | 9.461(8.728-10.28) |  |  |
|  | DEL | 20 | 7.301(6.022-8.202) | 0.004 | UCEC |
|  | GAIN | 17 | 8.974(7.925-9.688) |  |  |
|  | No Change | 503 | 8.243(7.155-9.356) |  |  |
| HYOU1 | DEL | 32 | 12.645(12.047-13.508) | 0.018 | BLCA |
|  | GAIN | 15 | 13.601(13.077-14.096) |  |  |
|  | No Change | 361 | 12.949(12.345-13.568) |  |  |
|  | DEL | 158 | 13.037(12.498-13.565) | < 0.001 | BRCA |
|  | GAIN | 23 | 14.105(13.374-14.598) |  |  |
|  | No Change | 909 | 13.318(12.812-13.863) |  |  |
|  | DEL | 50 | 12.361(11.892-12.782) | < 0.001 | CESC |
|  | GAIN | 2 | 14.201(13.473-14.929) |  |  |
|  | No Change | 242 | 12.927(12.367-13.518) |  |  |
|  | DEL | 8 | 13.308(13.106-13.732) | 0.005 | ESCA |
|  | GAIN | 4 | 14.702(14.47-15.208) |  |  |
|  | No Change | 149 | 13.975(13.428-14.362) |  |  |
|  | DEL | 59 | 12.945(12.337-13.459) | 0.006 | HNSC |
|  | GAIN | 9 | 13.601(13.359-14.261) |  |  |
|  | No Change | 428 | 13.214(12.692-13.857) |  |  |
|  | DEL | 4 | 12.513(12.179-12.667) | 0.016 | LGG |
|  | GAIN | 21 | 13.445(12.818-13.772) |  |  |
|  | No Change | 503 | 12.893(12.519-13.328) |  |  |
|  | DEL | 21 | 12.685(11.852-12.958) | < 0.001 | LIHC |
|  | GAIN | 6 | 14.223(13.97-14.349) |  |  |
|  | No Change | 345 | 13.209(12.558-13.87) |  |  |
|  | DEL | 27 | 12.976(12.328-13.654) | < 0.001 | LUAD |
|  | GAIN | 23 | 14.184(13.737-14.889) |  |  |
|  | No Change | 474 | 13.528(12.886-14.094) |  |  |
|  | DEL | 30 | 13.278(12.737-13.667) | < 0.001 | LUSC |
|  | GAIN | 20 | 14.567(14.192-14.938) |  |  |
|  | No Change | 450 | 13.776(13.262-14.213) |  |  |
|  | DEL | 44 | 13.201(12.605-13.524) | < 0.001 | OV |
|  | GAIN | 41 | 14.137(13.398-14.724) |  |  |
|  | No Change | 292 | 13.48(12.826-14.11) |  |  |
|  | DEL | 34 | 13.319(12.55-13.93) | 0.004 | PRAD |
|  | GAIN | 6 | 13.474(13.17-14.213) |  |  |
|  | No Change | 456 | 13.869(13.205-14.49) |  |  |
|  | DEL | 33 | 12.175(11.705-12.392) | < 0.001 | SARC |
|  | GAIN | 7 | 13.095(12.745-13.805) |  |  |
|  | No Change | 222 | 12.556(12.106-13.123) |  |  |
|  | DEL | 73 | 12.984(12.664-13.578) | < 0.001 | SKCM |
|  | GAIN | 3 | 14.582(14.429-14.99) |  |  |
|  | No Change | 394 | 13.596(12.929-14.17) |  |  |
|  | DEL | 20 | 13.306(12.874-13.924) | 0.004 | STAD |
|  | GAIN | 9 | 14.76(14.126-15.297) |  |  |
|  | No Change | 344 | 13.611(13.088-14.224) |  |  |
|  | DEL | 11 | 12.736(12.132-13.176) | 0.044 | TGCT |
|  | GAIN | 1 | 12.735(12.735-12.735) |  |  |
|  | No Change | 144 | 13.555(12.706-14.334) |  |  |
|  | DEL | 18 | 12.366(11.609-12.784) | < 0.001 | UCEC |
|  | GAIN | 18 | 13.975(12.782-14.24) |  |  |
|  | No Change | 504 | 12.711(12.065-13.349) |  |  |
| HSPA8 | DEL | 23 | 14.465(13.966-15.072) | 0.02 | LIHC |
|  | GAIN | 3 | 15.312(15.254-15.895) |  |  |
|  | No Change | 346 | 15.047(14.334-15.514) |  |  |
|  | DEL | 50 | 15.937(15.253-16.283) | 0.016 | OV |
|  | GAIN | 33 | 16.261(15.845-17.023) |  |  |
|  | No Change | 294 | 15.909(15.196-16.366) |  |  |
|  | DEL | 72 | 16.188(15.697-16.55) | 0.01 | SKCM |
|  | GAIN | 4 | 17.036(16.886-17.242) |  |  |
|  | No Change | 394 | 16.337(15.707-16.758) |  |  |
| HSP90B1 | DEL | 70 | 15.334(15.019-15.826) | < 0.001 | BRCA |
|  | GAIN | 24 | 16.214(15.617-16.721) |  |  |
|  | No Change | 996 | 15.631(15.229-16.086) |  |  |
|  | DEL | 5 | 15.068(15.009-15.292) | 0.041486 | CHOL |
|  | No Change | 31 | 15.691(15.192-15.814) |  |  |
|  | DEL | 6 | 14.312(14.108-15.264) | 0.015 | LGG |
|  | GAIN | 5 | 15.191(15.181-15.403) |  |  |
|  | No Change | 517 | 14.506(14.177-14.83) |  |  |
|  | DEL | 10 | 15.116(14.741-15.427) | 0.033 | LIHC |
|  | GAIN | 9 | 15.867(15.807-16.034) |  |  |
|  | No Change | 353 | 15.675(15.168-16.188) |  |  |
|  | DEL | 20 | 15.476(15.1-15.886) | 0.023 | LUSC |
|  | GAIN | 5 | 16.626(16.175-17.157) |  |  |
|  | No Change | 475 | 15.831(15.381-16.223) |  |  |
|  | DEL | 55 | 15.06(14.509-15.683) | < 0.001 | OV |
|  | GAIN | 16 | 16.063(15.422-16.437) |  |  |
|  | No Change | 306 | 15.508(15.02-15.989) |  |  |
|  | DEL | 3 | 14.413(14.334-14.592) | 0.022869 | PCPG |
|  | No Change | 165 | 15.252(14.925-15.647) |  |  |
|  | DEL | 6 | 14.849(14.548-15.119) | 0.001 | SARC |
|  | GAIN | 20 | 15.747(15.431-16.249) |  |  |
|  | No Change | 236 | 15.345(14.955-15.746) |  |  |
|  | DEL | 7 | 14.897(14.463-15.329) | 0.003916 | TGCT |
|  | No Change | 149 | 15.754(15.343-16.253) |  |  |
| HSPH1 | DEL | 27 | 12.617(12.131-12.893) | < 0.001 | BLCA |
|  | GAIN | 39 | 13.652(13.144-14.274) |  |  |
|  | No Change | 342 | 12.869(12.312-13.545) |  |  |
|  | DEL | 70 | 12.836(12.133-13.458) | < 0.001 | BRCA |
|  | GAIN | 89 | 13.805(13.04-14.394) |  |  |
|  | No Change | 931 | 13.056(12.458-13.667) |  |  |
|  | DEL | 45 | 12.55(12.035-12.953) | 0.002 | CESC |
|  | GAIN | 4 | 13.321(12.806-13.98) |  |  |
|  | No Change | 245 | 13.006(12.43-13.502) |  |  |
|  | DEL | 9 | 13.702(12.696-13.899) | 0.003 | COAD |
|  | GAIN | 21 | 13.561(12.556-14.032) |  |  |
|  | No Change | 432 | 12.598(11.579-13.505) |  |  |
|  | DEL | 10 | 10.997(10.196-11.494) | 0.001 | GBM |
|  | GAIN | 2 | 11.945(11.743-12.146) |  |  |
|  | No Change | 152 | 12.156(11.704-12.558) |  |  |
|  | DEL | 54 | 13.057(12.289-13.552) | < 0.001 | HNSC |
|  | GAIN | 10 | 13.968(13.664-14.305) |  |  |
|  | No Change | 432 | 13.398(12.749-14.024) |  |  |
|  | DEL | 43 | 10.884(10.27-11.58) | 0.024 | LIHC |
|  | GAIN | 7 | 11.545(11.191-12.425) |  |  |
|  | No Change | 322 | 11.266(10.675-11.873) |  |  |
|  | DEL | 43 | 12.843(12.203-13.285) | < 0.001 | LUSC |
|  | GAIN | 19 | 13.474(13.305-14.244) |  |  |
|  | No Change | 438 | 12.915(12.419-13.448) |  |  |
|  | DEL | 43 | 12.124(11.56-12.8) | < 0.001 | OV |
|  | GAIN | 29 | 13.198(12.711-14.091) |  |  |
|  | No Change | 305 | 12.492(11.957-12.985) |  |  |
|  | DEL | 32 | 11.48(10.815-12.05) | < 0.001 | PRAD |
|  | GAIN | 12 | 12.357(11.804-12.645) |  |  |
|  | No Change | 452 | 12.02(11.549-12.483) |  |  |
|  | DEL | 8 | 12.267(11.285-12.659) | 0.004 | READ |
|  | GAIN | 17 | 13.875(12.934-14.626) |  |  |
|  | No Change | 140 | 12.859(11.862-13.855) |  |  |
|  | DEL | 35 | 11.273(10.71-11.807) | < 0.001 | SARC |
|  | GAIN | 27 | 12.42(11.668-12.93) |  |  |
|  | No Change | 200 | 12.064(11.431-12.684) |  |  |
|  | DEL | 14 | 13.237(12.352-13.806) | < 0.001 | STAD |
|  | GAIN | 30 | 13.903(13.565-14.214) |  |  |
|  | No Change | 329 | 13.045(12.436-13.663) |  |  |
| HSP90AA1 | DEL | 55 | 16.185(15.821-16.787) | < 0.001 | BRCA |
|  | GAIN | 61 | 17.376(16.725-17.92) |  |  |
|  | No Change | 974 | 16.327(15.719-16.817) |  |  |
|  | DEL | 16 | 15.657(15-16.077) | 0.002 | CESC |
|  | GAIN | 7 | 16.552(16.416-16.993) |  |  |
|  | No Change | 271 | 16.034(15.592-16.579) |  |  |
|  | DEL | 11 | 15.79(15.497-16.668) | 0.006 | ESCA |
|  | GAIN | 9 | 17.155(16.958-17.419) |  |  |
|  | No Change | 141 | 16.504(16.104-16.962) |  |  |
|  | DEL | 20 | 15.121(14.702-15.695) | < 0.001 | HNSC |
|  | GAIN | 22 | 16.146(15.996-16.776) |  |  |
|  | No Change | 454 | 16.065(15.559-16.519) |  |  |
|  | DEL | 11 | 14.42(13.977-14.812) | 0.034 | KIRC |
|  | GAIN | 1 | 15.779(15.779-15.779) |  |  |
|  | No Change | 518 | 14.975(14.516-15.354) |  |  |
|  | DEL | 28 | 15.207(14.893-15.362) | < 0.001 | LGG |
|  | GAIN | 1 | 16.561(16.561-16.561) |  |  |
|  | No Change | 499 | 15.776(15.407-16.156) |  |  |
|  | DEL | 23 | 14.337(13.799-14.831) | < 0.001 | LIHC |
|  | GAIN | 2 | 14.795(14.504-15.087) |  |  |
|  | No Change | 347 | 15.011(14.559-15.572) |  |  |
|  | DEL | 29 | 15.202(14.305-15.718) | 0.002 | LUAD |
|  | GAIN | 18 | 16.28(15.289-17.269) |  |  |
|  | No Change | 477 | 15.467(14.902-16.121) |  |  |
|  | DEL | 13 | 15.325(15.139-15.539) | < 0.001 | LUSC |
|  | GAIN | 58 | 16.65(16.241-17.028) |  |  |
|  | No Change | 429 | 16.016(15.455-16.483) |  |  |
|  | DEL | 28 | 15.171(14.741-15.642) | < 0.001 | OV |
|  | GAIN | 74 | 16.263(15.679-16.957) |  |  |
|  | No Change | 275 | 15.673(15.229-16.144) |  |  |
|  | DEL | 5 | 14.866(14.536-14.999) | 0.015 | PCPG |
|  | GAIN | 1 | 16.131(16.131-16.131) |  |  |
|  | No Change | 162 | 15.65(15.192-15.977) |  |  |
|  | DEL | 10 | 14.845(14.253-15.111) | < 0.001 | PRAD |
|  | GAIN | 4 | 16.215(15.818-16.55) |  |  |
|  | No Change | 482 | 15.645(15.222-16.07) |  |  |
|  | DEL | 7 | 14.904(14.573-15.189) | < 0.001 | SARC |
|  | GAIN | 23 | 15.845(15.526-16.442) |  |  |
|  | No Change | 232 | 15.129(14.691-15.775) |  |  |
|  | DEL | 19 | 15.442(15.005-15.763) | 0.004 | SKCM |
|  | GAIN | 2 | 17.029(16.987-17.07) |  |  |
|  | No Change | 449 | 16.184(15.576-16.77) |  |  |
|  | DEL | 20 | 15.72(15.179-16.132) | < 0.001 | STAD |
|  | GAIN | 15 | 17.099(16.367-17.36) |  |  |
|  | No Change | 338 | 16.125(15.568-16.565) |  |  |
|  | DEL | 8 | 14.684(14.478-14.984) | < 0.001 | UCEC |
|  | GAIN | 35 | 16.067(14.937-16.745) |  |  |
|  | No Change | 497 | 14.892(14.162-15.728) |  |  |
| TRAP1 | DEL | 56 | 10.92(10.319-11.618) | < 0.001 | BLCA |
|  | GAIN | 5 | 11.306(9.494-12.44) |  |  |
|  | No Change | 347 | 11.394(10.916-11.911) |  |  |
|  | DEL | 19 | 11.488(10.595-11.634) | < 0.001 | BRCA |
|  | GAIN | 62 | 12.323(11.818-12.757) |  |  |
|  | No Change | 1009 | 11.949(11.467-12.388) |  |  |
|  | DEL | 2 | 10.629(10.572-10.687) | 0.009 | CESC |
|  | GAIN | 20 | 12.289(11.671-12.781) |  |  |
|  | No Change | 272 | 11.868(11.317-12.306) |  |  |
|  | DEL | 12 | 11.535(10.982-11.874) | < 0.001 | HNSC |
|  | GAIN | 15 | 12.585(11.934-13.157) |  |  |
|  | No Change | 469 | 11.855(11.448-12.313) |  |  |
|  | DEL | 27 | 11.667(11.162-12.02) | < 0.001 | LUSC |
|  | GAIN | 7 | 12.848(12.46-13.658) |  |  |
|  | No Change | 466 | 11.908(11.349-12.296) |  |  |
|  | DEL | 39 | 11.854(11.375-12.209) | < 0.001 | OV |
|  | GAIN | 20 | 12.869(12.38-13.462) |  |  |
|  | No Change | 318 | 12.345(11.747-12.849) |  |  |
|  | DEL | 5 | 10.512(10.289-10.942) | 0.018 | SARC |
|  | GAIN | 15 | 11.299(11.107-11.881) |  |  |
|  | No Change | 242 | 11.16(10.628-11.582) |  |  |
|  | DEL | 8 | 11.282(11.085-11.469) | < 0.001 | SKCM |
|  | GAIN | 16 | 12.474(12.218-12.777) |  |  |
|  | No Change | 446 | 12.048(11.656-12.541) |  |  |
|  | DEL | 23 | 11.56(11.133-11.892) | < 0.001 | STAD |
|  | GAIN | 11 | 12.471(11.544-12.948) |  |  |
|  | No Change | 339 | 12.135(11.635-12.636) |  |  |
|  | DEL | 17 | 10.66(9.394-10.827) | 0.004 | UCEC |
|  | GAIN | 8 | 12.044(11.508-12.28) |  |  |
|  | No Change | 515 | 11.031(10.442-11.717) |  |  |
| HSPA12B | DEL | 64 | 8.283(7.861-8.994) | < 0.001 | BRCA |
|  | GAIN | 47 | 8.527(7.335-9.136) |  |  |
|  | No Change | 979 | 8.994(8.304-9.638) |  |  |
|  | DEL | 15 | 7.358(6.538-7.89) | 0.011 | HNSC |
|  | GAIN | 10 | 6.57(6.037-7.174) |  |  |
|  | No Change | 471 | 7.607(6.87-8.331) |  |  |
|  | DEL | 3 | 7.295(6.925-7.339) | 0.039453 | MESO |
|  | No Change | 83 | 8.271(7.5-9.063) |  |  |
|  | DEL | 11 | 7.679(7.375-8.099) | < 0.001 | UCEC |
|  | GAIN | 29 | 7.827(7.055-8.504) |  |  |
|  | No Change | 500 | 6.775(6.044-7.48) |  |  |
| HSPA13 | DEL | 18 | 9.753(9.255-10.122) | 0.004 | BLCA |
|  | GAIN | 26 | 10.648(10.26-11.112) |  |  |
|  | No Change | 364 | 10.092(9.465-10.676) |  |  |
|  | DEL | 68 | 10.838(10.256-11.288) | < 0.001 | BRCA |
|  | GAIN | 47 | 11.889(11.357-12.505) |  |  |
|  | No Change | 975 | 11.285(10.713-11.86) |  |  |
|  | DEL | 15 | 9.96(9.81-10.17) | 0.004 | CESC |
|  | GAIN | 3 | 10.97(10.782-12.106) |  |  |
|  | No Change | 276 | 10.514(9.911-11.016) |  |  |
|  | DEL | 13 | 10.777(10.55-11.155) | 0.002 | ESCA |
|  | GAIN | 9 | 12.076(11.691-12.39) |  |  |
|  | No Change | 139 | 11.324(10.892-11.737) |  |  |
|  | DEL | 9 | 9.731(9.329-10.196) | < 0.001 | HNSC |
|  | GAIN | 27 | 11.11(10.508-11.632) |  |  |
|  | No Change | 460 | 10.442(9.838-11.004) |  |  |
|  | DEL | 27 | 10.211(9.488-11.101) | < 0.001 | LUAD |
|  | GAIN | 26 | 12.077(10.99-12.331) |  |  |
|  | No Change | 471 | 10.979(10.344-11.535) |  |  |
|  | DEL | 24 | 10.716(10.08-11.232) | < 0.001 | LUSC |
|  | GAIN | 39 | 11.945(11.253-12.409) |  |  |
|  | No Change | 437 | 10.981(10.386-11.639) |  |  |
|  | DEL | 38 | 10.276(9.232-10.691) | < 0.001 | OV |
|  | GAIN | 39 | 10.853(10.558-11.61) |  |  |
|  | No Change | 300 | 10.875(10.172-11.318) |  |  |
|  | DEL | 6 | 9.322(8.846-9.687) | 0.001549 | PAAD |
|  | No Change | 171 | 10.403(9.878-10.957) |  |  |
|  | DEL | 10 | 10.469(9.575-11.343) | 0.011 | SKCM |
|  | GAIN | 10 | 12.014(11.733-12.051) |  |  |
|  | No Change | 450 | 11.418(10.616-12.002) |  |  |
|  | DEL | 22 | 9.901(9.667-10.557) | < 0.001 | STAD |
|  | GAIN | 12 | 11.34(11.024-11.741) |  |  |
|  | No Change | 339 | 10.999(10.406-11.52) |  |  |
|  | DEL | 13 | 9.535(8.459-10.349) | 0.004 | UCEC |
|  | GAIN | 11 | 11.109(9.886-11.631) |  |  |
|  | No Change | 516 | 9.465(8.777-10.186) |  |  |
|  | DEL | 4 | 10.484(10.23-10.86) | 0.025 | UCS |
|  | GAIN | 5 | 11.88(11.789-12.525) |  |  |
|  | No Change | 46 | 11.067(10.826-11.794) |  |  |
| HSPA6 | DEL | 2 | 9.116(8.612-9.62) | < 0.001 | BLCA |
|  | GAIN | 100 | 9.186(8.103-10.59) |  |  |
|  | No Change | 306 | 8.248(7.51-9.147) |  |  |
|  | DEL | 19 | 8.124(7.768-8.897) | < 0.001 | BRCA |
|  | GAIN | 156 | 8.553(7.705-9.555) |  |  |
|  | No Change | 915 | 8.028(7.18-8.932) |  |  |
|  | GAIN | 71 | 9.008(7.974-10.058) | 0.021679 | LUAD |
|  | No Change | 453 | 8.558(7.693-9.502) |  |  |
|  | DEL | 1 | 7.672(7.672-7.672) | 0.015 | LUSC |
|  | GAIN | 57 | 9.292(8.455-10.394) |  |  |
|  | No Change | 442 | 8.815(8.062-9.587) |  |  |
|  | GAIN | 9 | 8.922(8.234-10.934) | 0.005417 | PAAD |
|  | No Change | 168 | 7.827(7.053-8.625) |  |  |
|  | GAIN | 3 | 8.29(7.899-8.826) | 0.037972 | THCA |
|  | No Change | 504 | 6.794(5.901-7.604) |  |  |
| N represented sample size. | |  |  |  |  |

| **Table S6 Associations of HSPA2 expression with clinicopathological parameters in Gastric cancer** | | | | |
| --- | --- | --- | --- | --- |
|  |  | **HSPA2 expression** | |  |
| **Variables** | **Cases** | **Low n (%)** | **High n (%)** | ***P* value** |
| Gender | 50 |  |  |  |
| Male | 35 | 20(57.1) | 15(42.9) |  |
| Female | 15 | 5(33.3) | 10(66.7) | 0.123 |
| Age(years) | 51 |  |  |  |
| >60 | 21 | 12(57.1) | 9(42.9) |  |
| <=60 | 30 | 13(43.3) | 17(56.7) | 0.332 |
| Smoking | 35 |  |  |  |
| Never Smoker | 26 | 13(50.0) | 13(50.0) |  |
| Ever Smoker | 9 | 6(66.7) | 3(33.3) | 0.387 |
| Drinking | 35 |  |  |  |
| Nondrinker | 28 | 13(46.4) | 15(53.6) |  |
| Drinker | 7 | 6(85.7) | 6(14.3) | 0.062 |
| Lauren classification | 50 |  |  |  |
| Intestinal | 7 | 5(71.4) | 2(28.6) |  |
| Diffuse | 38 | 18(47.4) | 20(52.6) |  |
| Mixed | 5 | 2(40.0) | 3(60.0) | 0.618 |
| Depth of invasion | 49 |  |  |  |
| Mucous and submucosal layer | 26 | 14(53.8) | 12(46.2) |  |
| Muscular and subserosa layer | 10 | 7(70.0) | 3(30.0) |  |
| Serosal layer or invasion adjacent organs | 4 | 0(0.0) | 4(100.0) | 0.159 |
| Lymph node metastasis | 47 |  |  |  |
| Positive | 27 | 16(59.3) | 11(40.7) |  |
| Negative | 20 | 7(35) | 13(65) | 0.142 |
| TNM stage | 47 |  |  |  |
| Ⅰ+Ⅱ | 27 | 18(59.3) | 11(40.7) |  |
| Ⅲ+Ⅳ | 20 | 7(35.0) | 13(65.0) | 0.142 |

| **Table S7 Associations of HSPA2, HSPA1A and HSPA7 expression with clinicopathological parameters in Colorectal cancer** | | | | | | | | | | | | | | |
| --- | --- | --- | --- | --- | --- | --- | --- | --- | --- | --- | --- | --- | --- | --- |
|  |  | **HSPA2 expression** | |  |  |  | **HSPA1A expression** | |  |  |  | **HSPA7 expression** | |  |
| **Variables** | **Cases** | **Low n (%)** | **High n (%)** | ***P* value** |  | **Cases** | **Low n (%)** | **High n (%)** | ***P* value** |  | **Cases** | **Low n (%)** | **High n (%)** | ***P* value** |
| Gender | 41 |  |  |  |  | 42 |  |  |  |  | 39 |  |  |  |
| Male | 24 | 12(50) | 12(50) |  |  | 22 | 13(59.1) | 9(40.9) |  |  | 21 | 10(47.6) | 11(52.4) |  |
| Female | 17 | 8(52.9) | 9(47.1) | 0.853 |  | 20 | 8(40.0) | 12(60.0) | 0.217 |  | 18 | 9(50.0) | 9(50.0) | 1.000 |
| Age(years) | 41 |  |  |  |  | 42 |  |  |  |  | 39 |  |  |  |
| >60 | 23 | 13(56.5) | 10(43.5) |  |  | 22 | 11(50.0) | 11(50.0) |  |  | 22 | 11(50.0) | 11(50.0) |  |
| <=60 | 18 | 7(38.9) | 11(61.1) | 0.262 |  | 20 | 10(50.0) | 10(50.0) | 1.000 |  | 17 | 8(47.1) | 9(52.9) | 1.000 |
| Smoking | 41 |  |  |  |  | 42 |  |  |  |  | 39 |  |  |  |
| Never Smoker | 31 | 14(45.2) | 17(54.8) |  |  | 32 | 15(46.9) | 17(53.1) |  |  | 29 | 14(48.3) | 15(51.7) |  |
| Ever Smoker | 10 | 6(60.0) | 4(40.0) | 0.651 |  | 10 | 6(60.0) | 4(40.0) | 0.719 |  | 10 | 5(50.0) | 5(50.0) | 1.000 |
| Drinking | 41 |  |  |  |  | 42 |  |  |  |  | 39 |  |  |  |
| Nondrinker | 31 | 15(48.4) | 16(51.6) |  |  | 33 | 14(42.4) | 19(57.6) |  |  | 29 | 13(44.8) | 16(55.2) |  |
| Drinker | 10 | 5(50.0) | 5(50.0) | 0.929 |  | 9 | 7(77.8) | 2(22.2) | 0.133 |  | 10 | 6(60.0) | 4(40.0) | 0.480 |
| Family history | 41 |  |  |  |  | 42 |  |  |  |  | 39 |  |  |  |
| Yes | 6 | 4(66.7) | 2(33.3) |  |  | 6 | 3(50.0) | 3(50.0) |  |  | 5 | 4(80.0) | 1(20.0) |  |
| No | 35 | 16(45.7) | 19(54.3) | 0.612 |  | 36 | 18(50.0) | 18(50.0) | 1.000 |  | 34 | 15(44.1) | 19(55.9) | 0.182 |
| Maximum diameter (cm) | 39 |  |  |  |  | 40 |  |  |  |  | 38 |  |  |  |
| >4 | 23 | 14(60.9) | 9(39.1) |  |  | 24 | 14(58.3) | 10(41.7) |  |  | 22 | 9(40.9) | 13(59.1) |  |
| <=4 | 16 | 6(37.5) | 10(62.5) | 0.151 |  | 16 | 6(37.5) | 10(62.5) | 0.197 |  | 16 | 9(56.3) | 7(43.8) | 0.512 |
| Lymph node metastasis | 41 |  |  |  |  | 42 |  |  |  |  | 39 |  |  |  |
| Positive | 18 | 9(50.0) | 9(50.0) |  |  | 19 | 9(47.4) | 10(52.6) |  |  | 18 | 8(44.4) | 10(55.6) |  |
| Negative | 23 | 11(47.8) | 12(52.2) | 0.890 |  | 23 | 12(52.2) | 11(47.8) | 0.757 |  | 21 | 11(52.4) | 10(47.6) | 0.751 |
| Differentiation degree | 41 |  |  |  |  | 42 |  |  |  |  | 39 |  |  |  |
| Well/moderate | 29 | 14(48.3) | 15(51.7) |  |  | 11 | 7(63.6) | 4(36.4) |  |  | 11 | 6(54.5) | 5(45.5) |  |
| Poor/mucinous | 12 | 6(50.0) | 6(50.0) | 0.920 |  | 31 | 14(45.2) | 17(54.8) | 0.292 |  | 28 | 13(46.4) | 15(53.6) | 0.731 |
| Growth pattern | 41 |  |  |  |  | 42 |  |  |  |  | 39 |  |  |  |
| Infiltrative | 21 | 12(47.6) | 11(52.4) |  |  | 23 | 12(52.2) | 11(47.8) |  |  | 21 | 11(57.9) | 10(47.6) |  |
| Nested/cloddy | 20 | 10(50.0) | 10(50.0) | 0.879 |  | 19 | 9(47.4) | 10(52.6) | 0.757 |  | 18 | 8(44.4) | 10(55.6) | 0.751 |
| Lymphatic/venous invasion | 41 |  |  |  |  | 42 |  |  |  |  | 39 |  |  |  |
| Positive | 4 | 1(25.0) | 3(75.0) |  |  | 6 | 4(66.7) | 2(33.3) |  |  | 5 | 3(60.0) | 2(40.0) |  |
| Negative | 37 | 19(51.4) | 18(48.6) | 0.635 |  | 36 | 17(47.2) | 19(52.8) | 1.000 |  | 34 | 16(47.1) | 18(52.9) | 0.661 |
| Invasive extent | 41 |  |  |  |  | 42 |  |  |  |  | 39 |  |  |  |
| T1-2 | 15 | 5(33.3) | 10(66.7) |  |  | 16 | 6(37.5) | 10(62.5) |  |  | 25 | 12(48.0) | 13(52.0) |  |
| T3-4 | 26 | 15(57.7) | 11(42.3) | 0.133 |  | 26 | 15(57.7) | 11(42.3) | 0.204 |  | 14 | 7(50.0) | 7(50.0) | 1.000 |
| TNM stage | 41 |  |  |  |  | 42 |  |  |  |  | 39 |  |  |  |
| Ⅰ+Ⅱ | 22 | 10(45.5) | 12(54.5) |  |  | 22 | 11(50.0) | 11(50.0) |  |  | 19 | 9(48.7) | 10(52.6) |  |
| Ⅲ+Ⅳ | 19 | 10(52.6) | 9(47.4) | 0.647 |  | 20 | 10(50.0) | 10(50.0) | 1.000 |  | 20 | 10(50.0) | 10(50.0) | 1.000 |
